# Pharmacogenetic stimulation of neuronal activity increases myelination in an axon-specific manner

**DOI:** 10.1038/s41467-017-02719-2

**Published:** 2018-01-22

**Authors:** Stanislaw Mitew, Ilan Gobius, Laura R. Fenlon, Stuart J. McDougall, David Hawkes, Yao Lulu Xing, Helena Bujalka, Andrew L. Gundlach, Linda J. Richards, Trevor J. Kilpatrick, Tobias D. Merson, Ben Emery

**Affiliations:** 1 The Florey Institute of Neuroscience and Mental Health, Parkville, VIC 3052 Australia; 2 Department of Anatomy & Neuroscience, The University of Melbourne, Parkville, VIC 3010 Australia; 3 Australian Regenerative Medicine Institute, Monash University, Clayton, VIC 3800 Australia; 4 Queensland Brain Institute, The University of Queensland, St Lucia, QLD 4072 Australia; 5 Department of Pharmacology and Therapeutics, The University of Melbourne, Parkville, VIC 3010 Australia; 6 Jungers Center for Neurosciences Research, Department of Neurology, Oregon Health and Science University, Portland, OR 97239 USA; 7 Florey Department of Neuroscience and Mental Health, The University of Melbourne, Parkville, VIC 3010 Australia; 8 Schools of Biomedical Sciences, The University of Queensland, St Lucia, QLD 4072 Australia; 9 Melbourne Neuroscience Institute, The University of Melbourne, Parkville, VIC 3010 Australia

## Abstract

Mounting evidence suggests that neuronal activity influences myelination, potentially allowing for experience-driven modulation of neural circuitry. The degree to which neuronal activity is capable of regulating myelination at the individual axon level is unclear. Here we demonstrate that stimulation of somatosensory axons in the mouse brain increases proliferation and differentiation of oligodendrocyte progenitor cells (OPCs) within the underlying white matter. Stimulated axons display an increased probability of being myelinated compared to neighboring non-stimulated axons, in addition to being ensheathed with thicker myelin. Conversely, attenuating neuronal firing reduces axonal myelination in a selective activity-dependent manner. Our findings reveal that the process of selecting axons for myelination is strongly influenced by the relative activity of individual axons within a population. These observed cellular changes are consistent with the emerging concept that adaptive myelination is a key mechanism for the fine-tuning of neuronal circuitry in the mammalian CNS.

## Abstract (web summary)

Neuronal activity is known to increase myelination by oligodendrocytes in the mouse brain. Here, Mitew et al. demonstrate that chemogenetic manipulations of somatosensory axon activity both increase the generation of new oligodendrocytes and preferentially enhance myelination of the activated axons.

## Introduction

The myelination of axons by oligodendrocytes is a critical step in central nervous system (CNS) development, facilitating action potential transmission and providing axons with metabolic and trophic support^1, 2^. While the caliber of an axon directly influences the probability of myelination, since even synthetic nanorods of a diameter >0.4 µm are myelinated by oligodendrocytes in vitro^3, 4^, additional factors appear to dictate which axons are myelinated in vivo. In certain white matter tracts, such as the optic nerve, virtually all axons are myelinated by adulthood^5^, whereas in other tracts, only a subpopulation of axons are myelinated, despite being of sufficient diameter. For example, in the corpus callosum (CC) there is considerable overlap in the diameters of unmyelinated and myelinated axons^6^. Hence, additional mechanisms regulating axonal selection for myelination must exist.

One exciting possibility is that neuronal activity could influence myelination in an adaptive manner, allowing for strengthening or synchronization of specific connections and circuits. Functional imaging studies in humans have shown that training for a specific skill, such as juggling or practicing a musical instrument, results in structural changes in the underlying white matter circuits that are associated with performing these tasks^7, 8^. The structural changes reported in these studies are presumably associated with the increased level and/or type of activity within these circuits and could be due to either the addition of newly generated myelin or the remodeling of existing myelin sheaths^9^. Recent evidence from rodent studies suggest that white matter structural changes, also potentially associated with increased myelination, are directly proportional to the level of performance in a motor task^10^ and indicate that motor learning requires the addition of newly generated oligodendrocytes^11^.

An open question is the extent to which an oligodendrocyte response to neuronal activity is specifically targeted to the axons that initiated it. At the cellular level, neuronal activity could theoretically lead to a direct (and potentially highly specific) increase in the probability of the activated axons being selected for myelination. Alternatively, axonal activity could augment the proliferation of oligodendrocyte progenitor cells (OPCs) or the generation of oligodendrocytes resulting in an increase in the myelination of nearby axons in a relatively non-selective manner. A variety of model systems provide evidence for both possibilities. For example, both in vitro and in vivo studies have demonstrated that increasing neuronal activity positively regulates OPC proliferation^12–14^ resulting in increased production of oligodendrocytes and myelin^12^. Other studies have shown that blocking neuronal activity has the opposite effect, decreasing OPC proliferation^15, 16^. In addition to these paracrine effects on local OPC proliferation, recent evidence indicates that neuronal activity directly influences axonal selection for myelination such that differentiating oligodendrocytes preferentially myelinate electrically active axons. In support of this, blocking vesicular release in a subset of axons in neuronal cultures^17^ or in the developing zebrafish nervous system^18, 19^ reduces the proportion of these axons that are selected for myelination. However, these studies on nerve populations do not assess the issue of specificity of axonal selection in the intact mammalian CNS.

As the relative contribution of the direct and indirect activity-dependent myelination programs could have substantial implications for the ability to modify circuits, we sought to determine whether activation of a subset of neurons would lead to either a broad or specific increase in myelination in the postnatal mouse brain. We demonstrate that non-invasive, pharmacogenetic stimulation of neuronal activity robustly increases the proliferation and differentiation of OPCs in the CC of both juvenile and adult mice, resulting in an increased density of newly differentiated oligodendrocytes capable of myelinating axons. The myelin generated is preferentially targeted to stimulated axons, resulting in a substantial increase in the proportion of activated axons that are myelinated relative to the total axon population. These results demonstrate that neuronal activity potentiates OPC proliferation within the white matter and selectively enhances myelination of active axons within the tract.

## Results

### Stimulation of neuronal activity in vivo

**Fig. 1:**
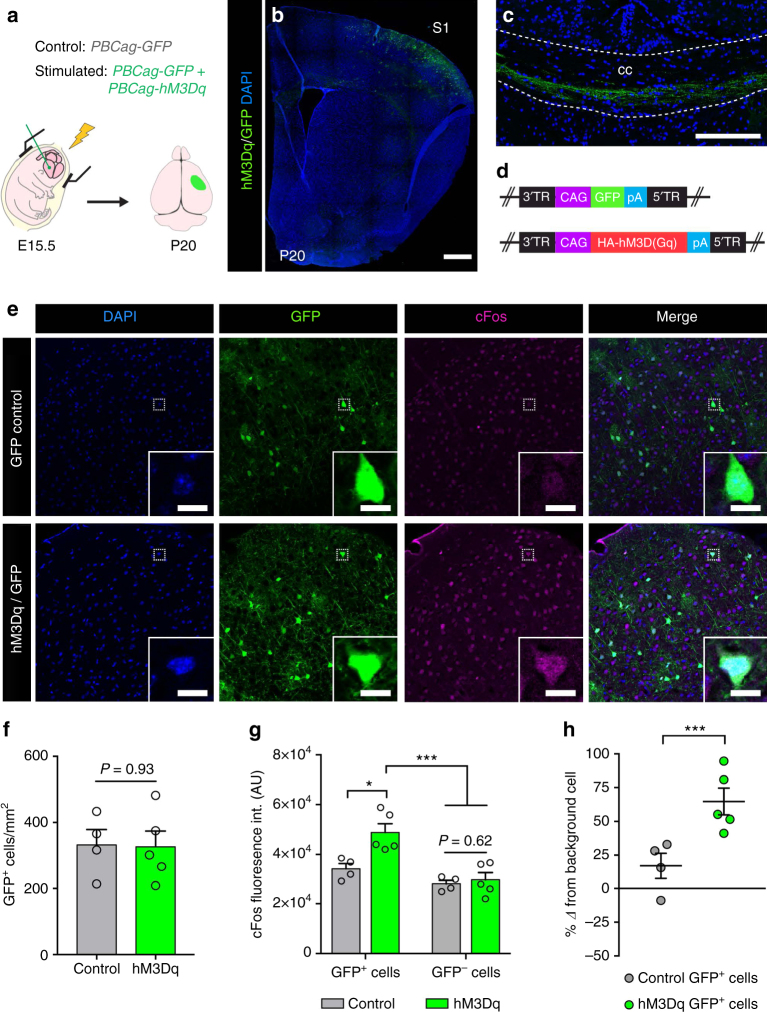
DREADD-mediated neuronal stimulation results in increased cFos expression. **a**–**c** *PBCag-GFP* (GFP control) or a 1:1 combination of *PBCag-GFP* and *PBCag-hM3Dq* (hM3Dq/GFP) were electroporated unilaterally into the right cerebral neuroepithelium of CD1 mice at E15.5 (**a**), resulting in a highly stereotypical and reproducible expression pattern in layer 2/3 S1 pyramidal neurons (**b**) and their axonal projections in the corpus callosum at P20 (**c**). **d** Structure of the PiggyBac plasmids encoding *hM3Dq* and *Gfp* that were used. **e** Representative images showing cFos expression in the S1 cortex of GFP control (top panel) and hM3Dq/GFP (bottom panel) mice following a week of CNO administration (P19–26). **f** Plasmid electroporation efficiency was similar between hM3Dq/GFP and GFP control mice. **g** The normalized absolute intensity of cFos immunostaining was significantly increased in hM3Dq/GFP electroporated neurons compared to that of GFP controls or non-electroporated neurons following a week of CNO-mediated stimulation (AU, arbitrary units). **h** Graph depicting the percentage change in cFos normalized absolute intensity from the average GFP^−^ cell intensity within samples. Welch’s corrected unpaired two-tailed *t*-test: *P < 0.05, ***P < 0.001; *n* = 6 mice/group,  ± s.e.m. Scale bars = 500 µm (**b**), 200 µm (**c**,** e**), 10 µm (insets, **e**). See also Supplementary Fig. 1

To modulate neuronal activity, we utilized the hM3Dq DREADD (designer receptor exclusively activated by designer drug), which causes depolarization of neurons in response to the synthetic ligand clozapine-*N*-oxide (CNO) or its metabolite clozapine^20, 21^. Specifically, we singly electroporated *Gfp* (GFP control) or co-electroporated *HA-hM3Dq* and *Gfp* plasmids (hM3Dq/GFP) unilaterally into the right cortical neuroepithelium in utero at E15.5 (Fig. 1a–d). This co-electroporation approach gave a high degree of co-expression; within the hM3Dq/GFP group, roughly 60% of GFP^+^ transfected neurons had detectable HA-hM3Dq^+^ expression while ~90% of the HA-hM3Dq^+^ neurons co-expressed GFP (Supplementary Fig. 1). The *Gfp* plasmid had a comparable transfection efficacy between GFP control and hM3Dq/GFP groups (Fig. 1e, f). In vivo, CNO administration resulted in increased cFos expression in hM3Dq/GFP mice relative to GFP controls (Fig. 1g, h). In addition, we also confirmed that CNO directly mediates depolarization and action potential firing in hM3Dq-expressing neurons by performing whole-cell patch clamp recordings of transduced pyramidal neurons in cortical slices (Supplementary Fig. 2). As previously described for hippocampal neurons^20^, incubation of P20 acute brain slice preparations with 1 µM CNO resulted in an average resting membrane depolarization of 6.5 ± 0.5 mV in hM3Dq^+^ neurons (Supplementary Fig. 2h, i). Collectively, these data demonstrate that CNO administration evokes a specific and robust activation response in neurons expressing the hM3Dq receptor.

### Neuronal stimulation of OPC proliferation and differentiation

**Fig. 2:**
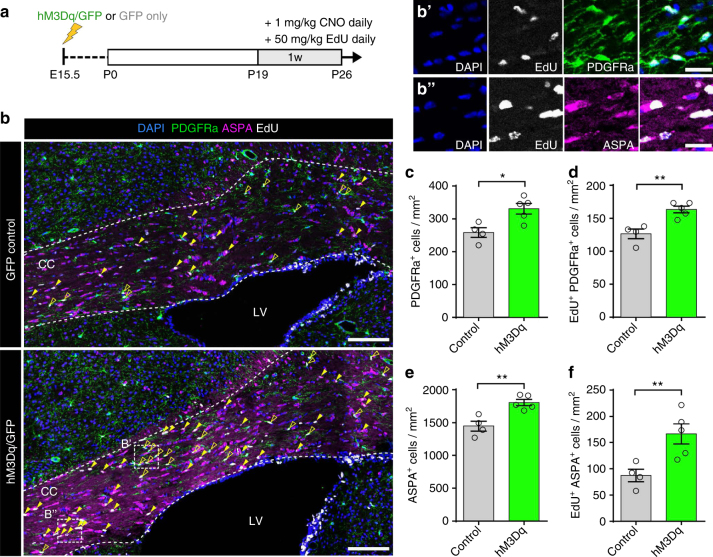
Activity stimulation enhances oligodendrogenesis in juvenile mice. **a** Experimental timeline illustrating onset of CNO/EdU administration in juvenile mice that had been electroporated with plasmids containing either *hM3Dq* and *Gfp* (hM3Dq) or *Gfp* only (Control) in utero at E15.5. **b** Coronal sections through the corpus callosum at P19+7 were immunolabeled with OPC (PDGFRa) and mature oligodendrocyte (ASPA) markers and the number of proliferating (EdU^+^) OPCs (open arrowheads, **b’**) and newly differentiated oligodendrocytes (filled arrowheads, **b”**) was quantified. **c**–**f** Activity stimulation resulted in increased levels of progenitor cell proliferation (**c**,** d**), as well as enhanced differentiation and oligodendrogliogenesis (**e**, **f**). Welch’s corrected unpaired two-tailed *t*-test: **P < 0.01, *P < 0.05; *n* = 4 Control mice (GFP only), *n* = 5 hM3Dq/GFP mice, ± s.e.m. Scale bars = 200 µm (**b**), 20 µm (**b’**, **b”**). See also Supplementary Figs. 2, 3

We assessed the proliferative response of OPCs in the CC following chronic pharmacogenetic stimulation of a small subset of callosal axons. Specifically, we co-administered CNO and 5’-ethynyl-2’-deoxyuridine (EdU) to P19 hM3Dq/GFP or GFP control mice for 7 consecutive days prior to perfusion fixation (P19+7 mice; Fig. 2a). P19+7 mice expressing hM3Dq/GFP displayed an overall 28.1 ± 8.7% increase in the density of PdgfRα+OPCs (Fig. 2b; *P* = 0.014, control: *n* = 4 mice and hM3Dq: *n* = 5 mice, Welch’s corrected *t*-test) as well as a 29.2 ± 6.7% increase in the density of mitotically active EdU^+^/PdgfRα^+^ OPCs (*P* = 0.003, control: *n* = 4 mice and hM3Dq: *n* = 5 mice, Welch’s corrected *t*-test) relative to green fluorescent protein (GFP) controls, indicative of increased levels of OPC proliferation and/or survival (Fig. 2c, d). Additionally, in the hM3Dq/GFP group there was an overall increase in the density of cells labeled with the mature oligodendrocyte marker ASPA (25.0 ± 6.0%, *P* = 0.004, control: *n* = 4 mice and hM3Dq: *n* = 5 mice, Welch’s corrected *t*-test) in the region of the CC encompassing the hM3Dq^+^ axons (Fig. 2e). This increase in oligodendrocyte density was likely due to enhanced OPC differentiation, as hM3Dq/GFP mice displayed a 90.6 ± 27.6% increase in the density of EdU^+^/ASPA^+^ cells (Fig. 2b, b”) compared to GFP controls (Fig. 2f, *P* = 0.014, control: *n* = 4 mice and hM3Dq: *n* = 5 mice, Welch’s corrected *t*-test).

We conducted the same experiment using a viral approach (infection of the somatosensory cortex with AAV1/2-CAG-hM3Dq-mCherry, Supplementary Fig. 3a, b) and observed very similar effects on OPC proliferation and oligodendrocyte differentiation after 1 week of stimulation (Supplementary Fig. 3c–f). In contrast, a shorter stimulation period of 24 h did not lead to a significant change in the density of total or cycling OPCs (Supplementary Fig. 3g, h). The proliferative effect seen after 1 week of stimulation was specific to the oligodendroglial lineage with no change detected in the density or proportion of dividing astrocytes (EdU^+^/GFAP^+^) or microglia (EdU^+^/Iba1^+^) (Supplementary Fig. 4). This indicates that the OPC proliferative effects were not part of a pathophysiological response to stimulation.

**Fig. 3:**
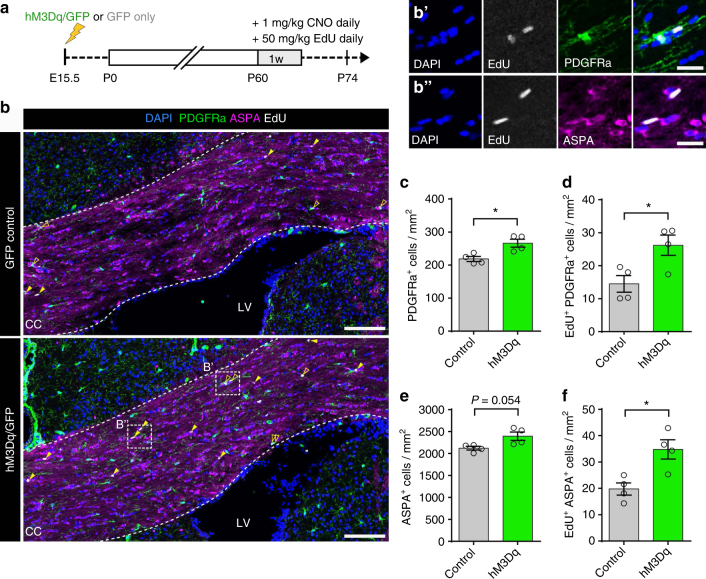
Activity stimulation in adult mice results in increased oligodendrogenesis. **a** Experimental timelime indicating onset of CNO/EdU administration in adult mice that had been electroporated with plasmids containing either *hM3Dq* and *Gfp* (hM3Dq) or *Gfp* only (Control) in utero at E15.5. **b** Coronal sections through the corpus callosum at P60+14 were immunolabeled with OPC (PDGFRa) and mature oligodendrocyte (ASPA) markers and the number of proliferating (EdU^+^) OPCs (open arrowheads, **b’**) and newly differentiated oligodendrocytes (filled arrowheads, **b”**) was quantified. **c**–**f** Activity stimulation resulted in increased levels of OPC proliferation and cell cycle entry (**c**, **d**), which in turn resulted in increased EdU^+^ incorporation in adult ASPA^+^ cells (**f**), although the difference in overall number of mature oligodendrocytes was not significant between the groups (**e**). Welch’s corrected unpaired two-tailed *t*-test: *P < 0.05; *n* = 4 mice/group,  ± s.e.m. Scale bars = 200 µm (**b**), 20 µm (**b’**, **b”**). See also Supplementary Fig. 4

We next assessed whether pharmacogenetic stimulation was also sufficient to drive oligodendrogenesis in adult mice by co-administering CNO and EdU between P60 and P66. Interestingly, at P61 and P67 we detected no changes in EdU incorporation or OPC proliferation and differentiation (Supplementary Fig. 5a–d). To determine whether this could be due to the increased cell cycle time and delay between proliferation and differentiation with age^22, 23^, we extended the time of fixation to P74 (i.e., 2 weeks after the onset of stimulation) (Fig. 3a). At this extended time point, there was an 80.9 ± 27.4% (*P* = 0.027, *n* = 4 mice, Welch’s corrected *t*-test) increase in the density of dividing OPCs and a 21.8 ± 6.4% (*P* = 0.018, *n* = 4 mice, Welch’s corrected *t*-test) increase in the total density of PdgfRα^+^ OPCs in hM3Dq/GFP mice relative to GFP controls (Fig. 3b–d), indicating that stimulation also had an effect on OPC proliferation in the adult brain. There was also a strong trend toward an increase in the overall density of ASPA^+^ cells (Fig. 3e, *P* = 0.054, *n* = 4 mice, Welch’s corrected *t*-test) and a statistically significant 76.1 ± 21.8% increase in the density of newly differentiated EdU^+^/ASPA^+^ oligodendrocytes in response to neuronal activity stimulation at this later age (Fig. 3b, f, *P* = 0.017, *n* = 4 mice, Welch’s corrected *t*-test). These data confirm that the heightened activity increased the generation of new oligodendrocytes. Again, we observed very similar effects upon the oligodendroglial lineage in rAAV1/2-hM3Dq-mCherry-infected mice that were stimulated with CNO and assessed 2 weeks after the onset of stimulation (Supplementary Fig. 5f, g).

To explore the possibility that neuronal activity may alter the generation of oligodendrocytes through enhanced survival, we quantified the proportion of newly generated OPCs and oligodendrocytes that were targeted for apoptosis (Supplementary Fig. 6a, b). In juvenile mice, we observed a slightly lower proportion of cleaved caspase-3^+^ EdU^+^ OPCs in hM3Dq/GFP mice compared to controls (14.4 ± 5.4% decrease, GFP control vs. hM3Dq/GFP; *P* = 0.03, *n* = 5 mice, Welch’s corrected *t*-test; Supplementary Fig. 6c, d). Neuronal activity stimulation did not appear to promote survival of newly generated (EdU^+^) oligodendrocytes in juvenile mice (Supplementary Fig. 6e), however, in adult mice there was a slight trend for higher apoptosis in newly generated oligodendrocytes in hM3Dq/GFP mice compared to controls (*P* = 0.07, *n* = 4 mice, Welch’s corrected *t*-test; Supplementary Fig. 6f-h).

### Stimulation of neuronal activity increases myelination

Our data indicate that the pharmacogenetic activation of a subset of callosally projecting neurons leads to an increased pool of newly differentiated oligodendrocytes in the region encompassing the stimulated axons. As the CC contains a large number of axons that are potentially receptive to myelination, it is plausible that newly generated oligodendrocytes could either preferentially myelinate the stimulated axons or myelinate both the stimulated and non-stimulated axons in an indiscriminate manner.

**Fig. 4:**
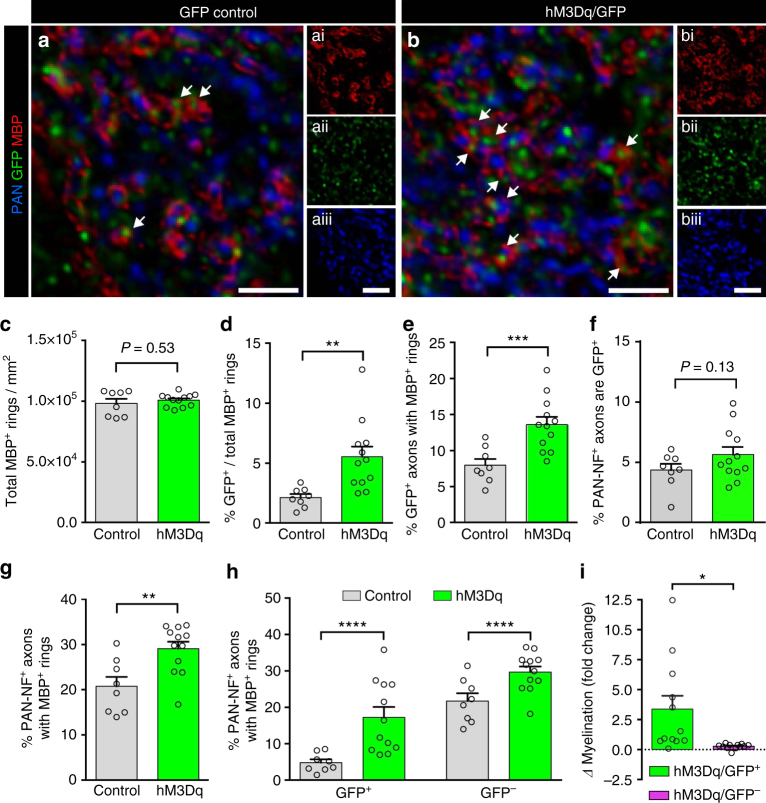
Activity stimulation results in increased myelination in an axon-selective manner. **a**, **b** Representative examples of GFP control (**a**) or hM3Dq/GFP (**b**) mouse callosal axons labeled with PAN-NF (blue), GFP (green), and MBP (red), with white arrows indicating myelinated GFP^+^ axons. **c**, **d** Although the total number of MBP^+^ rings in the corpus callosum was similar (**c**), the proportion of myelin rings with GFP^+^ axons was significantly higher in the hM3Dq-expressing mice compared to controls (**d**). **e** The percentage of GFP^+^ axons that colocalized with MBP^+^ rings was significantly higher in hM3Dq/GFP mice compared to GFP controls. **f** An equivalent percentage of PAN-NF^+^ axons co-expressed GFP in the hM3Dq/GFP and GFP control groups. **g** The percentage of PAN-NF^+^ axons that colocalized with MBP^+^ rings was higher in hM3Dq/GFP mice compared to GFP controls. **h** The percentage of GFP^+^ and GFP^–^ PAN-NF^+^ axons that were colocalized with MBP^+^ rings in each group. **i** The overall change in myelination in hM3Dq/GFP mice was much more pronounced in GFP^+^ axons compared to GFP^–^ axons (values computed as fold change from mean density of GFP^+/−^ PAN-NF^+^ MBP^+^ axons in GFP control mice). Welch’s corrected unpaired two-tailed *t*-test: ****P < 0.0001, ***P < 0.001, **P < 0.01, *P < 0.05; *n* = 8 Control mice (GFP only), *n* = 12 hM3Dq/GFP mice,  ± s.e.m. Scale bars = 2 µm (**a**, **b**)

To determine the effects of activity on target axon preference, hM3Dq/GFP and GFP control mice were administered CNO from P14–P20, corresponding to the peak period of myelin formation in the CC^6^. Sagittal sections of the CC were immunolabeled with antibodies against GFP to identify electroporated axons and myelin basic protein (MBP) to visualize myelin rings (Fig. 4a, b). Increasing neuronal activity did not alter the total density of MBP^+^ rings in the CC (Fig. 4c), however a higher percentage of these MBP^+^ rings was associated with GFP^+^ axons in hM3Dq/GFP mice compared to GFP control mice (Fig. 4d). Moreover, hM3Dq/GFP expressing axons were on average 1.7 times more likely to be surrounded by an MBP^+^ ring than were GFP control axons (Fig. 4e; *P* = 0.0007, control: *n* = 8 mice and hM3Dq: *n* = 12 mice, Welch’s corrected *t*-test).

To determine whether this increase in myelination was also present within the broader axonal population, we next examined the myelination status of all pan-neurofilament (PAN-NF) positive axons (detectable in ~30% of neurons in the rat neocortex and their projections^24^). We first confirmed that the percentage of PAN-NF^+^ axons that co-expressed GFP was similar in both GFP^−^ control and hM3Dq/GFP mice, reflecting comparable transduction efficiencies (4.3 ± 0.5% vs. 5.7 ± 0.6% respectively; Fig. 4f; *P* = 0.134, control: *n* = 8 mice and hM3Dq: *n* = 12 mice, Welch’s corrected *t*-test). Compared to GFP controls, a higher percentage of PAN-NF^+^ axons in hM3Dq/GFP mice was surrounded by MBP^+^ rings (Fig. 4g). To assess the specificity of the pro-myelinating effect of axonal stimulation in hM3Dq/GFP mice, we compared MBP ensheathment of both GFP^+^ PAN-NF^+^ axons and adjacent GFP^−^ PAN-NF^+^ axons that should not be directly activated by CNO, in comparison to GFP control mice (Fig. 4h). Interestingly, although there was a higher proportion of both GFP^+^ (17.2 ± 2.8% vs. 4.8 ± 0.9%; *P* < 0.0001, control: *n* = 8 mice and hM3Dq: *n* = 12 mice, two-way analysis of variance (ANOVA)) and GFP^–^ (29.7 ± 1.5% vs. 21.7 ± 2.1%; *P* < 0.0001, control: *n* = 8 mice and hM3Dq: *n* = 12 mice, two-way ANOVA) ensheathed PAN-NF^+^ axons in hM3Dq/GFP mice compared to GFP controls, the effect in GFP^+^ axons (~3.4-fold increase) was significantly more pronounced than in GFP^–^ axons (~0.3-fold increase) (Fig. 4i). These data suggest that elevating the activity of a subset of axons results in a predominantly axon-specific increase in myelination, with a smaller, potential “bystander” effect on surrounding non-stimulated axons within the microenvironment.

### Attenuation of neuronal activity reduces myelination

We have recently shown that Kir2.1 overexpression in cortical neurons results in a ~20 mV hyperpolarization, making them considerably less excitable^25^. Employing this approach, we reduced the activity of a subpopulation of transcallosal axons arising from the S1 area of cerebral cortex by co-electroporating plasmids containing *PBCag-Kir2.1* and *PBCag-Gfp* or *PBCag-Gfp* only in utero at E15.5. We then analyzed the myelination status of the electroporated axons in sagittal sections of the CC of P20 mice.

**Fig. 5:**
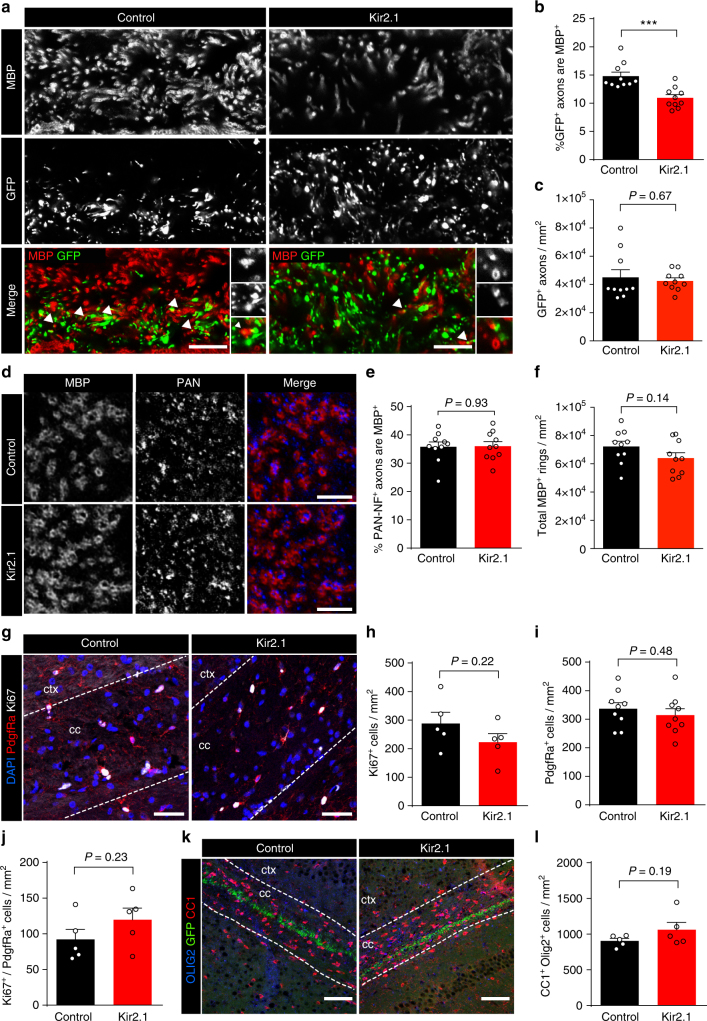
Inhibiting axonal activity during early development decreases myelination in an axon-selective manner. **a** Representative images of sagittal sections of the corpus callosum at the midline of P20 mice that had been electroporated with plasmids containing either *Kcnj2* and *Gfp* (Kir2.1) or *Gfp* only (Control) in utero at E15.5. White arrowheads indicate MBP^+^/GFP^+^ axons. **b**,** c** GFP^+^ axons in the Kir2.1-expressing mice displayed reduced myelination compared to GFP only electroporated controls (**b**), though the density of electroporated axons was comparable in both conditions (**c**). **d** Representative images showing MBP^+^/PAN-NF^+^ axons in the corpus callosum. **e**, **f** There was no difference in either the percentage of myelinated PAN-NF^+^ axons (**e**) or the density of MBP^+^ rings (**f**) between Kir2.1 mice and controls. **g** Representative images of the corpus callosum immunolabeled with OPC marker PdgfRa (red) and proliferation marker Ki67 (white). **h**–**j** There was no change in the density of proliferating cells (**h**), the total number of OPCs (**i**) or the density of proliferating OPCs (**j**) between Kir2.1 mice and controls. **k** Representative images of the corpus callosum immunolabeled with oligodendrocyte lineage marker OLIG2 (blue), mature oligodendrocyte marker CC1 (red), and GFP (green). **l** There was no significant difference in the density of mature oligodendrocytes between Kir2.1 and control mice. Welch’s corrected unpaired two-tailed *t*-test: ***P < 0.001; *n* = 10 mice/group (**a**–**f**), *n* = 5 mice/group (**g**–**l**),  ± s.e.m. Scale bars = 5 µm (**a**,** d**), 200 µm (**g**,** k**)

In contrast to our findings of hM3Dq-mediated neuronal activation, we observed that Kir2.1 overexpression repressed myelination, with the percentage of electroporated axons surrounded by MBP^+^ rings being 26.0 ± 6.1% lower in Kir2.1/GFP-overexpressing axons than for GFP-expressing control axons (Fig. 5a, b). The lower rate of Kir2.1/GFP axon myelination was not due to differences in electroporation efficiency and/or Kir2.1/GFP^+^ axon deficits at the midline (Fig. 5c). There was no difference in the density of MBP^+^/PAN^+^ axons (Fig. 5d, e) or the total density of MBP^+^ rings (Fig. 5f), suggesting that the decrease was specific to the reduced-activity axons. We then assessed whether this reduced level of myelination was the consequence of reduced oligodendrocyte differentiation or OPC proliferation (Fig. 5g). Constitutive Kir2.1 overexpression had no detectable effect on the density of dividing (Ki67^+^) cells (Fig. 5h), PdgfRα^+^ OPCs (Fig. 5i), proliferating OPCs (Fig. 5j), or mature CC1^+^ oligodendrocytes (Fig. 5k, l). These data strongly suggest that the observed decrease in the proportion of Kir2.1-expressing axons that were myelinated is mediated at the level of axonal selection rather than altered oligodendrocyte production.

### Neuronal activity stimulates radial myelin sheath growth

**Fig. 6:**
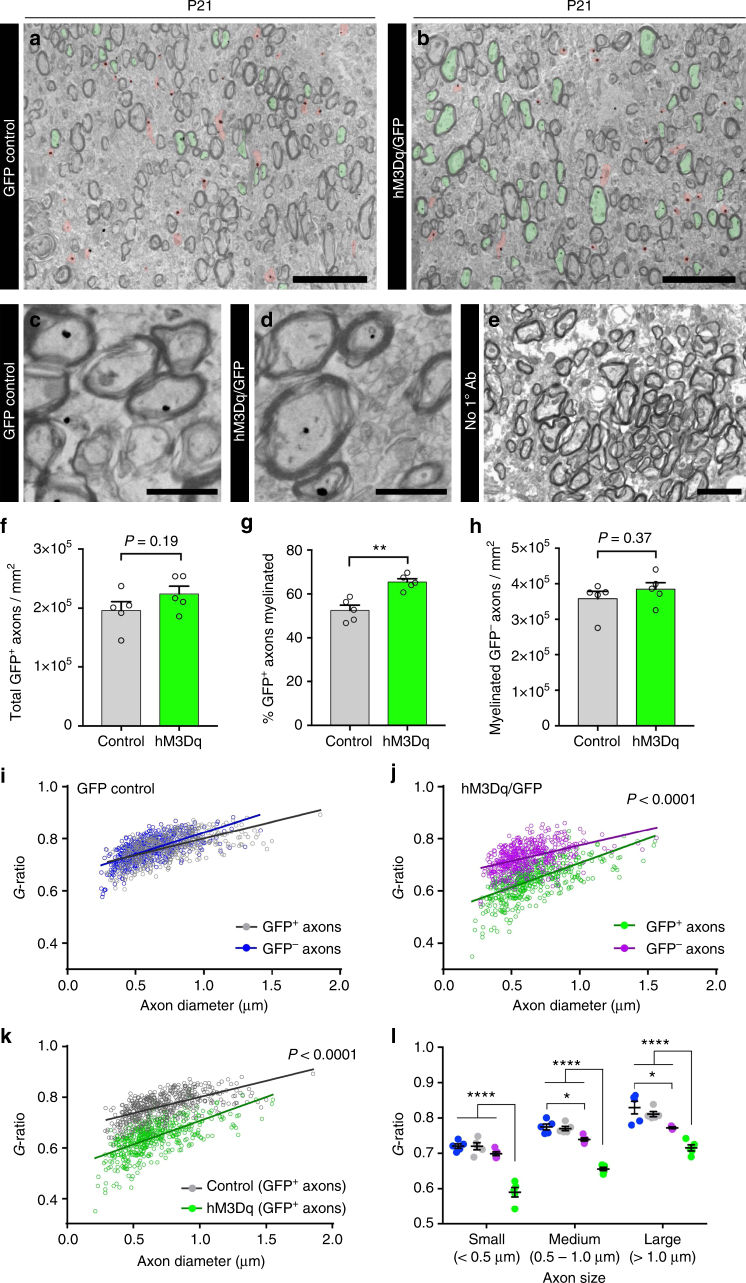
Stimulation of neuronal activity in juvenile mice promotes myelination and results in thicker myelin sheaths. **a**,** b** Representative immunogold electron microscopic images of the corpus callosum of GFP control (**a**) and hM3Dq/GFP (**b**) mice at P21, following a week of CNO administration (P14–20). Myelinated and unmyelinated GFP^+^ axons are pseudo-colored green and red, respectively. **c**, **d** Higher magnification of immunogold-positive axons from GFP control (**c**) and hM3Dq/GFP (**d**) mice. **e** No primary antibody control sections had almost no gold deposits. **f** Analysis of the density of GFP^+^ axons in each condition. **g** Quantification of the percentage of GFP^+^ axons myelinated in each condition; hM3Dq/GFP mice displayed a significant increase in the percentage of GFP^+^ axons that were myelinated compared to GFP only control mice (Welch’s corrected unpaired two-tailed *t*-test: **P < 0.01, *n* = 5 mice/condition). **h** Quantification of the density of myelinated GFP^–^ axons in each condition. **i**,** j** Analysis of myelin thickness (*g*-ratios) for GFP^+^ and GFP^–^ axons in GFP control (**i**) and hM3Dq/GFP (**j**) mice. **k** Comparison of the *g*-ratios of the GFP^+^ population in GFP control and hM3Dq/GFP mice. **l** Breakdown of *g*-ratios for each group by axonal size. Colors of conditions matched for **i**–**k**. Two-way ANOVA with Tukey’s multiple comparisons test: *P < 0.05, ****P < 0.0001, *n* = 5 mice/condition,  ± s.e.m. Scale bars = 5 µm (**a**,** b**), 1 µm (**c**,** d**), 2.5 µm (**e**). See also Supplementary Fig. 5

Adaptive myelination could be mediated not only by the presence or absence of myelin but also by regulating myelin thickness^26, 27^. We analyzed an independent cohort of CNO-treated P21 hM3Dq/GFP and GFP control electroporated mice by anti-GFP immunogold electron microscopy (EM). GFP-expressing axons were identified by the presence of one or more colloidal gold deposits within the axoplasm of callosal axons imaged in the sagittal plane (Fig. 6a–e). Consistent with our earlier light microscopic analysis (Fig. 4), there was a similar density of GFP^+^ axons in the control and hM3Dq/GFP groups (Fig. 6f) but an increase in the proportion of immunogold-labeled axons that were myelinated in hM3Dq/GFP electroporated mice compared to GFP controls (65.4 ± 1.5% vs. 52.5 ± 2.3%; Fig. 6g; *P* = 0.002, *n* = 5 mice, Welch’s corrected *t*-test). The density of myelinated GFP^−^ axons was similar between groups, suggesting that the myelination effect is preferential to the group of activated hM3Dq-expressing axons (Fig. 6h).

To assess whether increased neuronal activity during postnatal development altered the thickness of the myelin sheath surrounding myelinated axons, we measured *g*-ratios (ratio of axon caliber to total myelinated fiber caliber). As expected, there was no difference in the *g*-ratio of GFP^+^ and GFP^−^ axons in control mice (Fig. 6i). In contrast, the *g*-ratio of hM3Dq/GFP^+^ axons was markedly lower than that of adjacent (distance ≤ 10 µm) GFP^−^ axons (Fig. 6j), suggesting that, in addition to being selected for myelination more often, axons with heightened activity were also surrounded by thicker myelin sheaths. Likewise, when we compared the GFP^+^ axons in CNO-administered hM3Dq/GFP mice to CNO-administered GFP-only controls, the hM3Dq axons displayed significantly lower *g*-ratios, confirming that the effect was due to increased neuronal activity rather than an unrepresentative subset of axons being targeted by the electroporation (Fig. 6k).

Frequency distributions of the axonal diameter of myelinated GFP^+^ axons were similar for both hM3Dq/GFP and GFP control mice and closely reflected the size distribution of myelinated GFP^−^ axons within the CC (Supplementary Fig. 7a, b). Thus the effect of increasing myelin sheath thickness surrounding hM3Dq/GFP axons was not selective for axons of any particular diameter. The frequency distributions of myelinated axons expressed as a function of *g*-ratio revealed a clear shift in the distribution of hM3Dq/GFP^+^ axons toward lower *g*-ratios compared to GFP controls (Supplementary Fig. 7c). This was also confirmed when the mean *g*-ratios of small, medium, and large axons within the CC were examined, demonstrating a broad thickening of hM3Dq/GFP^+^ axon myelin sheaths across all axonal sizes (Fig. 6l). Interestingly, GFP^−^ axons in hM3Dq/GFP electroporated mice exhibited a modest shift in their distribution toward lower *g*-ratios (Supplementary Fig. 7c); however, this was predominantly restricted to medium and large axons (Fig. 6l). Collectively, these data reveal that, in addition to increasing the probability that an axon is selected for myelination, heightened neuronal activity results in an increase in the thickness of the myelin sheath surrounding active axons.

**Fig. 7:**
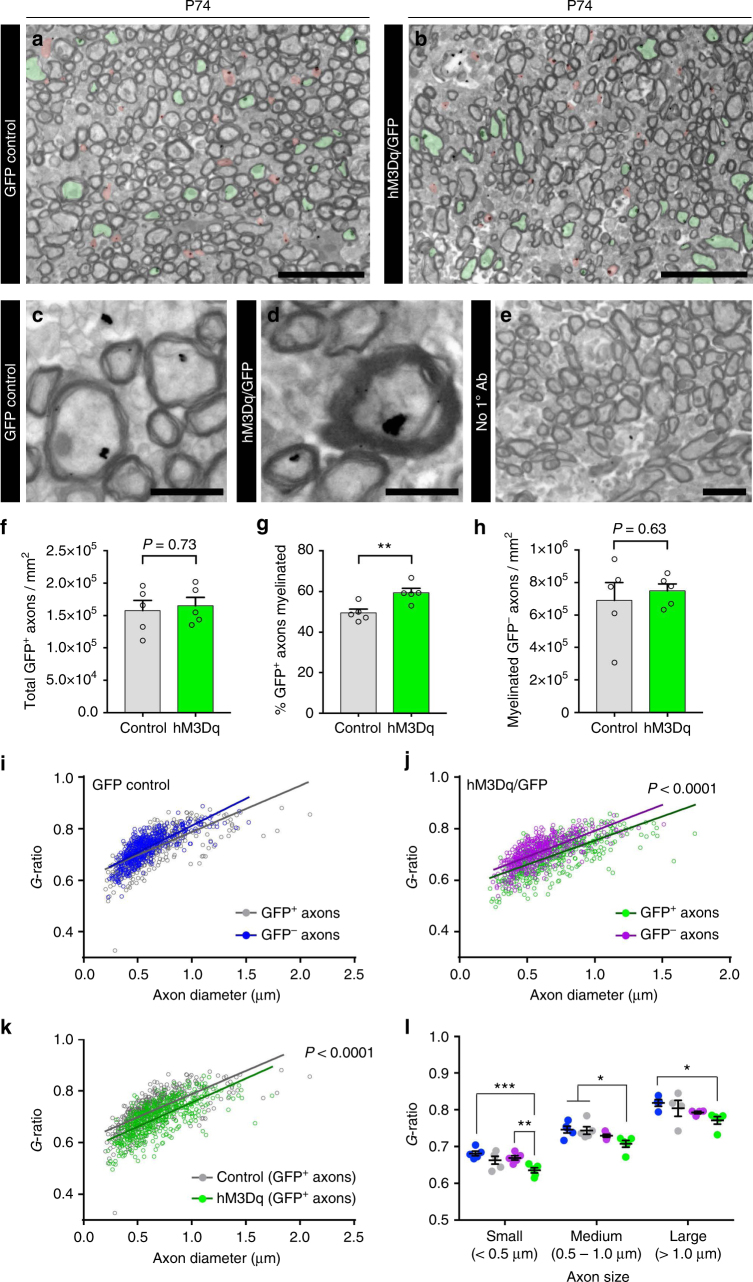
Stimulation of neuronal activity in adult mice promotes myelination and results in thicker myelin sheaths. **a**,** b** Representative immunogold electron microscopic images of the corpus callosum of GFP control (**a**) and hM3Dq/GFP (**b**) mice at P74, following a week of CNO administration (P60–66). Myelinated and unmyelinated GFP^+^ axons are pseudo-colored green and red, respectively. **c**, **d** Higher magnification of immunogold-positive axons from GFP control (**c**) and hM3Dq/GFP (**d**) mice. **e** No primary antibody control sections showed almost no gold deposits. **f** Analysis of the density of GFP^+^ axons in each condition. **g** Quantification of the percentage of GFP^+^ axons myelinated in each condition; hM3Dq/GFP mice displayed a significant increase in the percentage of GFP^+^ axons myelinated relative to GFP only control mice (Welch’s corrected unpaired two-tailed *t*-test: **P < 0.01, *n* = 5 mice/condition). **h** Quantification of the density of myelinated GFP^–^ axons in each condition. **i**,** j** Analysis of myelin thickness (*g*-ratios) for GFP^+^ and GFP^–^ axons in GFP control (**i**) and hM3Dq/GFP (**j**) mice. **k** Comparison of the *g*-ratios of the GFP^+^ population in GFP control and hM3Dq/GFP mice. **l** Breakdown of *g*-ratios for each group by axonal size. Colors of conditions matched for **i**–**k**. Two-way ANOVA with Tukey’s multiple comparisons test: *P < 0.05, **P < 0.01, ***P < 0.001; *n* = 5 mice/condition,  ± s.e.m. Scale bars = 5 µm (**a**, **b**), 1 µm (**c**, **d**), 2.5 µm (**e**). See also Supplementary Fig. 5

We repeated this experiment in adult mice to determine whether stimulating activity was still capable of altering myelination after adult steady-state myelination levels have been reached (Fig. 7a–e). Electroporated mice were stimulated with CNO from P60 to P66 then perfused at P74. We confirmed that both groups exhibited similar densities of GFP^+^ axons (myelinated and unmyelinated) indicating equivalent electroporation efficiencies (Fig. 7f). A higher percentage of GFP^+^ axons was myelinated in hM3Dq/GFP mice compared to GFP controls (hM3Dq/GFP 59.4 ± 2.1%, GFP control 49.5 ± 1.8%, Fig. 7g; *P* = 0.009, *n* = 5 mice, Welch’s corrected *t*-test). Importantly, the effect was restricted to the stimulated (GFP^+^) axons, as there was no change in the density of myelinated GFP^−^ axons (Fig. 7h). Axonal stimulation in adult mice also resulted in an increase in myelin thickness. While GFP^+^ and GFP^−^ axons had a very similar *g*-ratio distribution in non-stimulated control mice (Fig. 7i), stimulated GFP^+^ axons in hM3Dq-expressing mice had significantly lower *g*-ratios compared to either nearby GFP^−^ axons (Fig. 7j) or to control mice GFP^+^ axons (Fig. 7k, l; Supplementary Fig. 7f). The increase in the thickness of myelin that surrounded stimulated axons was observed irrespective of axonal diameter (Fig. 7l; Supplementary Fig. 7c, d).

### Preferential de novo myelination of stimulated axons

Although in theory the increased myelination of the activated axons could be provided by remodeling of existing myelin and/or increased branching of oligodendrocytes, the increase in EdU^+^/ASPA^+^ cells observed in the hM3Dq group suggested that preferential myelination of the activated axons by newly differentiated oligodendrocytes may mediate the effect. To directly test this hypothesis, we examined evidence for de novo myelination of stimulated callosal axons using *PdgfRα-CreER*^*T2*^*:Tau-mGFP* mice, which allow for the labeling of the myelin internodes of newly formed oligodendrocytes with membrane-targeted GFP^22^.

**Fig. 8:**
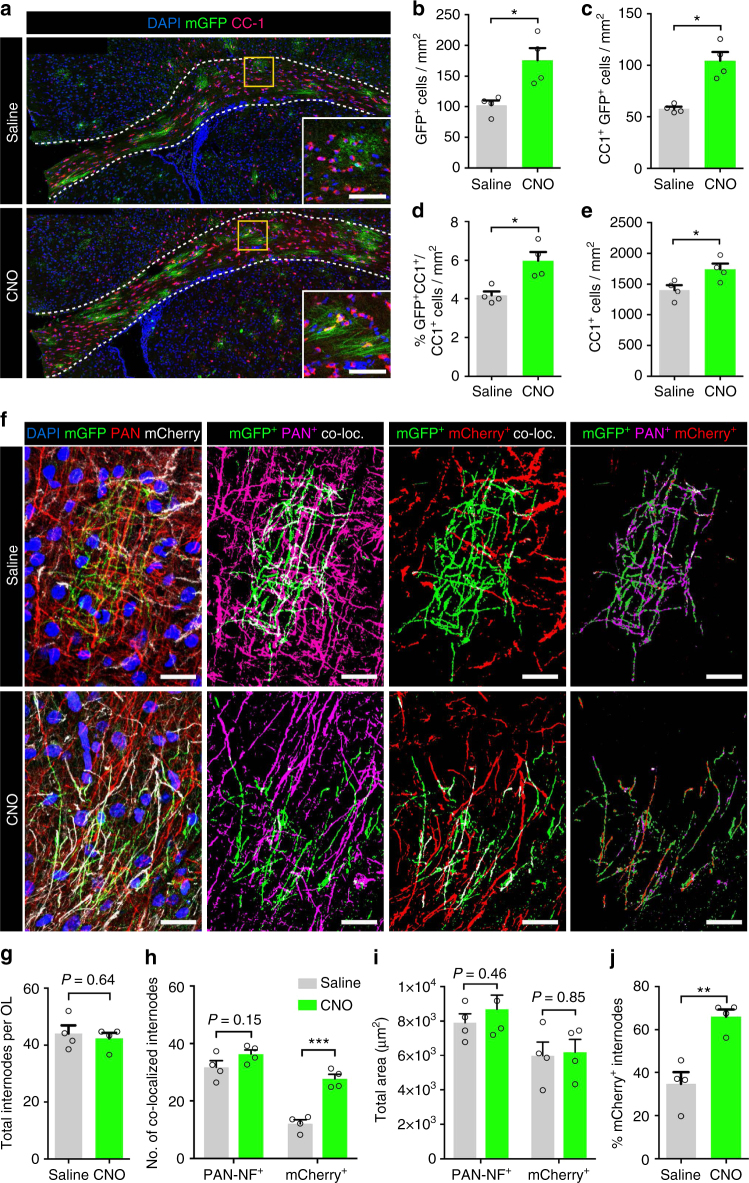
Newly generated oligodendrocytes preferentially target axons with higher levels of activity. **a** Representative images of the corpus callosum of *PdgfRα-CreERT*^*2*^*: Tau-mGFP* mice that had undergone 1 week of treatment with either CNO or saline. Coronal sections were immunolabeled with mature oligodendrocyte marker CC1 (magenta) and GFP (green); insets show a higher number of doubly labeled GFP^+^/CC1^+^ cells in CNO-treated mice (bottom inset) compared to saline controls (top inset). **b**, **c** Quantification of the density of GFP^+^ cells (**b**) and the density of CC1^+^/GFP^+^ cells (**c**) in the corpus callosum of CNO- or saline-treated mice. **d**, **e** Quantification of the percentage of all CC1^+^ cells that were GFP^+^ (**d**) and the total density of CC1^+^ cells (**e**) in stimulated and non-stimulated mice. **f** Representative images of newly differentiated cortical oligodendrocytes, demonstrating colocalization with PAN-NF^+^ or mCherry^+^ axons. **g** Analysis of the total number of internodes per oligodendrocyte. **h** Quantification of the number of internodes that were ensheathing PAN-NF^+^ or mCherry^+^ axons in each condition. **i** The total cortical area occupied by either PAN-NF^+^ or mCherry^+^ processes was similar in both CNO- and saline-treated mice. **j** The percentage of all internodes per oligodendrocytes that ensheathed mCherry^+^ axons was higher in CNO-treated mice compared to saline controls. Welch’s corrected unpaired two-tailed *t*-test: ***P < 0.001, **P < 0.01, *P < 0.05; *n* = 4 mice/condition, ± s.e.m. Scale bars = 200 µm (**a**), 40 µm (insets), 20 µm (**f**)

We injected the right S1 of *PdgfRα-CreER*^*T2*^*:Tau-mGFP* mice with the AAV1/2-CAG-hM3Dq-mCherry viral vector at P5 and treated them with tamoxifen at P18 to induce recombination of the *Tau-mGFP* reporter in OPCs. We then treated them from P19 to P26 with either saline or 1 mg/kg CNO. Consistent with our previous results, there was a significant increase of 72.2 ± 21.0% in the overall density of GFP^+^ cells in the activity-stimulated group compared to controls (Fig. 8a, b). This was accompanied by an 81.0 ± 15.1% increase in the density of GFP^+^ cells that had differentiated and acquired CC1 immunoreactivity (Fig. 8c), resulting in an increase in both the percentage of CC1^+^ cells that expressed GFP (Fig. 8d) and the total density of CC1^+^ cells (Fig. 8e).

To test whether the myelin from these newly generated oligodendrocytes was preferentially targeted to the activated axons, we analyzed the proportion of mGFP^+^ myelin processes that was associated with mCherry^+^ axons in CNO- or saline-treated controls (Fig. 8f). While the average number of mGFP^+^-labeled internodes per oligodendrocyte was similar between saline and CNO-administered mice (Fig. 8g), the number of internodes that were aligned with mCherry^+^ axons was more than two-fold higher in CNO-administered mice than in saline controls (12.1 ± 1.3 vs. 27.6 ± 1.5 for saline and CNO-administered mice, respectively; Fig. 8h; *P* = 0.003, *n* = 4 mice, Welch’s corrected *t*-test), despite the overall number of mCherry^+^ axons being similar in both groups (Fig. 8i). On average, 66.0 ± 3.3% of all GFP^+^ myelin internodes were associated with mCherry^+^ axons in CNO-administered mice compared to only 34.7 ± 5.4% in saline-treated controls (Fig. 8j). This result revealed a strong bias for newly differentiated oligodendrocytes to myelinate activated axons over neighboring axons in which activity was not modulated.

## Discussion

The ability to dynamically regulate myelination in response to environmental cues or neuronal activity is increasingly thought to be critical for normal brain function and learning^11, 12, 27, 28^. Apart from the myelination of previously unmyelinated axons, circuit function could also be modified by either altering the length of myelin internodes^29^ or the number of wraps (myelin thickness) on existing myelin^30^. Recent studies in rodents and zebrafish have raised the intriguing possibility that electrically active neurons may signal nearby OPCs to initiate differentiation and promote myelination^12, 18, 19^. However, the question arises as to how neuronal activity regulates myelination: are there generalized effects on the oligodendroglial lineage that impact the whole white matter tract or do individual axons compete for myelination? Our study demonstrates that neuronal activity enhances myelination in both juvenile and adult mice, albeit to a lesser degree in the latter. We determined that, in addition to promoting selective myelination of activated axons, stimulating activity increased myelin synthesis resulting in thicker myelin sheaths. This effect would be predicted to have a significant impact on overall circuit function as thicker myelin is associated with faster signal conduction^30^.

Broadly consistent with a previous study using optogenetic stimulation^12^, we observed a significant increase in OPC proliferation within the CC in response to 1 week of stimulation of a subset of somatosensory cortex neurons. Many of these proliferative OPCs went on to differentiate into mature oligodendrocytes, increasing the density of newly generated (EdU^+^) oligodendrocytes in the CC of juvenile and adult stimulated mice by 90.6% and 76.1%, respectively. While neuronal stimulation increased the density of OPCs and newly generated oligodendrocytes at both ages, the proliferation and differentiation of OPCs into mature oligodendrocytes was more protracted in adult mice than in juvenile mice (Supplementary Fig. 5), consistent with previous observations^22, 23, 31^. Interestingly, the increase in OPC density in juvenile mice appears to be mediated at least in part by a reduction in the percentage of OPCs that underwent apoptosis after cell division (Supplementary Fig. 6d). By contrast, the density of apoptotic (cleaved caspase-3^+^) OPCs in adult mice was an order of magnitude lower, was not detected among recently divided (EdU^+^) OPCs, and was unaffected by neuronal stimulation (Supplementary Fig. 6f, g). Given a 9.5-day cell cycle length of OPCs in the P60 CC^22^, we estimate that asymmetric division of OPCs in non-stimulated adult mice would generate ~300 postmitotic oligodendroglia/mm^2^ between P60 and P67, of which ~240 cells/mm^2^ survived to mature into ASPA^+^ oligodendrocytes by P74 (see Methods section for calculations). In stimulated mice, we calculate that ~420 new ASPA^+^ oligodendrocytes/mm^2^ were specified during the period of CNO administration, exceeding the number of postmitotic oligodendroglia that we estimate are generated under control conditions. Our finding that stimulated mice exhibited a ~49% increase in EdU incorporation rate among OPCs and a ~21% increase in OPC density support the possibility that increased OPC mitosis following neuronal stimulation is a prerequisite for potentiating oligodendrocyte production.

It is also entirely feasible that neuronal activity could enhance oligodendrocyte production by promoting OPCs to directly differentiate into oligodendrocytes without prior cell division. It has recently been demonstrated that providing mice with a motor learning task (complex wheel running) results in a rapid and robust enhancement in OPC differentiation into oligodendrocytes, with this preceding any detectable changes in OPC proliferation^11, 32^. In vivo live imaging studies similarly demonstrate that only a minority of OPC differentiation events are directly preceded by proliferation in the adult cerebral cortex^33^. Although an increase in OPC differentiation in the absence of proliferation would not be detected in our EdU incorporation experiments, it should be accounted for in the *PdgfRα-CreER*^*T2*^*:Tau-mGFP* lineage tracing experiments in which newly differentiated oligodendrocytes were genetically labeled with mGFP (Fig. 8a–c). These experiments showed an approximate doubling in the rate of oligodendrocyte differentiation with enhanced neuronal activity, an effect size that presumably incorporates the contributions of both OPC proliferation and direct differentiation into myelinating cells. Determining the exact sequence of these cellular events will be an important subject for ongoing studies, however.

Actively differentiating oligodendrocytes have a relatively limited time in which to survey their surroundings and select axons to myelinate^34, 35^. A recent zebrafish study reported that 75% of initial axon contacts by immature oligodendrocytes were unstable and quickly retracted and that individual axons in which vesicular release was blocked were less likely to be myelinated^18^. This supports the possibility that oligodendroglial processes that contact electrically active axons at putative axon–glia synapses^17, 36^ are more likely to be stabilized^18^ and therefore mature into myelin sheaths. Our findings that newly differentiated mGFP^+^ oligodendrocytes preferentially ensheath stimulated mCherry^+^ axons in CNO-administered mice, and that Kir2.1 expressing axons are less likely to be myelinated than their neighbors, indicate that levels of axonal activity bias their selection for myelination in the mammalian CNS as well. In mice expressing Kir2.1, the absence of detectable changes in OPC or oligodendrocyte density or overall myelination is also consistent with the effect being mediated at the level of axonal selection. Notably, we did not observe a change in the total internode number per oligodendrocyte between stimulated and non-stimulated mice. Although this somewhat differs from the findings of Mensch et al.^19^ (who found a decrease in internode number of oligodendrocytes when axonal vesicular release was blocked), our paradigm differs in that we are manipulating a comparatively small subset of axons within an already active population.

It is becoming increasingly evident that the effects of neuronal activity on myelination are likely to be contextual to the experimental paradigm, including the form of manipulation and the neuronal population studied. Etxeberria et al.^37^ recently reported that reducing axonal activity in the developing visual system through unilateral monocular deprivation or genetic blocking of retinal ganglion cell vesicular release did not decrease the proportion of axons that were myelinated in the optic nerve or tract but rather increased the number of newly differentiated oligodendrocytes with a concomitant decrease seen in the pool of proliferating OPCs. Given the optic nerve is essentially a fully myelinated tract, it seems possible that the increase in oligodendrocyte numbers reported in that study reflected a compensation for the reduced internode length seen in the deprived nerve/tract^37^. This also highlights the likelihood that activity will have different effects on myelination in different CNS regions either due to intrinsic differences in the regional pool of oligodendrocytes^38^ or the neuronal populations^39^.

The molecular and cellular mechanism(s) that underlie axonal selection by oligodendrocytes remain to be fully elucidated but are likely to include the above described activity-dependent vesicular release of neurotransmitters at axo-glial synapses^17, 36^. In addition, activity-dependent axonal selection could be mediated by differential expression of cell-surface ligands^40–42^. The relative contribution of vesicularly released and cell surface ligands in the process of axonal selection will be important to establish in future work.

Gibson et al.^12^ demonstrated an increase in myelin thickness in the subcortical white matter underlying the primary motor cortex that was optogenetically stimulated; however it was not possible to ascertain whether this was specific to the activated axons or whether it also extended to nearby unstimulated axons. By using immunogold EM to identity manipulated axons, we were able to definitively demonstrate that electrical stimulation resulted in a specific increase in myelin thickness on stimulated axons in the mammalian white matter compared to nearby unstimulated (GFP^−^) or GFP control axons, which had higher *g*-ratios. This thicker myelin membrane is not likely due to axonal caliber effects, as we identified a proportional thickening of myelin across all ranges of axon diameters, and the distribution of axonal diameters examined were very similar between the hM3Dq/GFP^+^ and GFP^+^ axons. Remarkably, this activity-mediated effect on myelin thickness was also present in adult mice, albeit not to the same extent as in juvenile mice. One interesting possibility in older mice is that, in addition to de novo myelination of unmyelinated axons, stimulation of myelinated axons may promote remodeling of their existing myelin. Such adaptations, dubbed myelin plasticity, could be used physiologically to fine-tune the conduction of action potentials, even in white matter tracts that are predominantly myelinated. Work by Snaidero et al.^43^ suggests this could occur through the opening of cytoplasmic channels within the myelin sheath in response to elevated phosphatidylinositol-(3,4,5)-triphosphate, allowing additional myelin membrane synthesis and radial growth. Other studies in mammalian cell culture^17^, in vivo zebrafish larvae^18, 39^, and in the developing mouse optic nerve^37^ suggest that neuronal activity also regulates myelin sheath length. Although we revealed that radial growth of myelin is stimulated by neuronal activity, we did not directly assess internode length in our studies.

In addition to the axon-specific effects following activity stimulation, we also observed small but significant effects on the percentage of GFP^–^ axons that were myelinated (Fig. 4g, h) and a slight decrease in the *g*-ratio of GFP^–^ axons in juvenile (Fig. 6l; Supplementary Fig. 7e) but not adult hM3Dq/GFP mice (Fig. 7l; Supplementary Fig. 7f). This suggests that while increasing activity within a tract principally stimulates myelination of active axons, myelination of non-stimulated axons could also be affected to some degree. We cannot, however, exclude the alternative possibility that some GFP^+^ axons were mis-classified as GFP^−^ (e.g., due to inefficiency of immunogold EM procedure) or that the difference is accounted for by the ~10% of HA-hM3Dq-expressing neurons that were GFP^−^ (Supplementary Fig. 1). Finally, recent evidence suggests that the CNO metabolite clozapine (rather than CNO itself) may be the direct DREADD ligand in vivo, raising the prospect of clozapine being present at sufficient levels to also act on endogenous receptors^21, 44^. This is unlikely to be a confounding factor in our study as both GFP control and hM3Dq/GFP mice received equal doses of CNO in the majority of experiments, allowing us to unequivocally attribute CNO effects to the DREADD-expressing cells.

In conclusion, we find that both paracrine effects on the turnover and differentiation of the oligodendroglial lineage in addition to more specific effects on axonal selection are relevant aspects of adaptive myelination in the postnatal mouse brain. In addition, electrical activity specifically regulates myelin thickness, an effect that persists into adulthood. These combined mechanisms likely play a critical role in fine-tuning axonal conductance according to the specific latencies required by different neural networks.

## Methods

### Transgenic mice

Animal experiments were performed in accordance with the National Health and Medical Research Council guidelines and were approved by the animal ethics committees of The Florey Institute of Neuroscience and Mental Health (Parkville, VIC, Australia) and The University of Queensland (Brisbane, QLD, Australia). CD1 mice used for in utero electroporation experiments were obtained from The University of Queensland Biological Resources Unit. C57Bl/6J mice used for rAAV1/2-sCAG-hM3Dq-mCherry experiments were obtained from the Animal Resources Centre (ARC, Western Australia). *PdgfRα-CreER*^*T2*^ transgenic mice^45^ were crossed to the *Tau-mGFP* reporter mouse line (denoted *PdgfRα-CreER*^*T2*^*:Tau-mGFP*), which results in mGFP expression in differentiating OPC processes^22^. Both the *PdgfRα-CreER*^*T2*^ and *Tau-mGFP* lines were maintained on a C57Bl/6J background. Recombination in *PdgfRα-CreER*^*T2*^*:Tau-mGFP* mice was induced 1 day before CNO treatment by oral gavage of tamoxifen (0.3 g/kg; Sigma-Aldrich Cat#T5648) prepared in corn oil (Sigma-Aldrich Cat#C8267). Both male and female mice were used in the study. Animals were assigned randomly to experimental groups. The number of mice assigned to each group was based on estimates of variance and the sample size required to identify a biologically relevant effect. Animal experiments were performed in an unblinded fashion; however, all quantitative data were collected with the investigator blinded to each group allocation (see subsection “Glial cell counts and myelin analysis”).

### In utero electroporation and plasmid preparation

We utilized the PiggyBac transposon system to allow stable transgene expression throughout life. Briefly, 0.5–1 µl of plasmid DNA (1 µg/µl) was injected into the right lateral ventricle of E15.5 CD1 embryos and electroporated into the S1 cortical neuroepithelium using five 100 ms pulses at 30 V delivered by external 3 mm paddle electrodes (Nepagene, Chiba, Japan) clamped across the uterus, which are connected to an ECM 830 electroporator (BTX Harvard Apparatus, Holliston, MA, USA). Plasmid expression constructs were pCAG-PBase and pPBCAG-GFP (kind gifts from Joseph LoTurco), pPBCAG-hM3Dq (subcloned from pcDNA5/FRT-HA-hM3D(Gq); Addgene #45547; into the pPBCAG vector backbone), and pCAG-Kir2.1 (kind gift from Yoshiaki Tagawa). Control GFP mice were electroporated with 1:1 ratio of *pCAG-PBase* and *pPBCAG-GFP*. hM3Dq/GFP mice were electroporated with 1:1:1 ratio of *pPBCAG-hM3Dq*, *pCAG-PBase*, and *pPBCAG-GFP*. Kir2.1/GFP mice were electroporated with a 1:1:1 ratio of *pCAG-Kir2.1*, *pCAG-PBase*, and *pPBCAG-GFP*. The E15.5 embryos of pregnant CD1 dams were electroporated at The University of Queensland and, following birth, the pups were transported to The University of Melbourne between P10 and P20 for CNO administration and further experimentation and analysis.

### In vivo CNO administration

CNO (Tocris Bioscience, Bristol, UK) was dissolved in 0.9% saline and was administered at 1 mg/kg chronically via intraperitoneal injection once daily starting at P19 or P60 for a week. At this dosage, the plasma half-life of CNO in mice is 2 h; however, owing to the robust binding of CNO or its metabolites to the hM3Dq receptor, the downstream effects persist for 6–10 h^20, 46^. For in utero electroporation experiments, control GFP mice received the same dose of CNO as the hM3Dq/GFP group. For AAV1/2-sCAG-hM3Dq-mCherry experiments, control mice received saline only. CNO treatment did not result in any gross behavioral changes or influence body weight.

### Tissue processing and immunohistochemistry

Mice were deeply anesthetized with 100 mg/kg sodium pentobarbitone and then perfused transcardially with phosphate-buffered saline (PBS), followed by 4% paraformaldehyde (PFA)/PBS. Brains were removed and postfixed in 4% PFA/PBS for 2 h on ice and cryopreserved in 30% sucrose/PBS overnight, followed by embedding in Tissue-Tek OCT compound (Sakura FineTek, Torrance, CA, USA). The brains were stored at −80 °C until sectioned on a Leica cryostat, collected onto Superfrost Plus slides (Menzel Glaser, Braunschweig, Germany), and air dried for 1 h before storing at −80 °C until stained. Sections of 10 µm thickness were obtained in the coronal and sagittal planes. Cryosections were blocked with PBS containing 0.3% Triton X-100, 10% normal donkey serum, and 0.01% sodium azide for 1 h at room temperature (RT). The sections were then incubated with primary antibodies at RT overnight, followed by 2 h incubation at RT with secondary antibody.

For cell fate and oligodendroglial population analysis, the following primary antibodies were used: mouse anti-APC/CC1 (1:300; Calbiochem Cat# OP80), goat anti-PdgfRα (1:150; R&D Systems Cat# AF1062), rabbit anti-ASPA (1:1000; gift from Dr Matthias Klugmann, UNSW), mouse anti-GFAP (1:200; Millipore Cat# MAB360), rabbit anti-Olig2 (1:100; IBL Cat# JP18953), rabbit anti-Iba1 (1:200; Wako Cat# 019-19741) mouse anti-NeuN (1:400; Millipore Cat# MAB377), chicken anti-GFP (1:2000; Abcam Cat# ab13970), rabbit anti-RFP (1:500; Rockland Cat# 600-401-379), mouse anti-parvalbumin (1:500; Sigma-Aldrich Cat# P3088), mouse anti-calretinin (1:200; Swant Cat# 6B3), rabbit anti-cFos (1:500; SantaCruz Cat# SC-52), mouse anti-Ki67 (MM1) (1:100; Novocastra Cat# PA0118), rat anti-HA high affinity (clone 3F10) (Sigma-Aldrich Cat# 11867423001), and rabbit anti-cleaved Caspase 3 antibody (Abcam Cat# ab2302).

For myelin analysis, the following primary antibodies were used: chicken anti-GFP (1:2000; Aves Labs Cat# GFP-1020), mouse anti-PAN (1:100; ThermoFisher Scientific Cat# 180171Z), and rat anti-MBP (1:300; Millipore Cat# MAB386). Secondary antibodies raised in donkey and conjugated to fluorescein isothiocyanate (FITC), TRITC, Alexa Fluor-555, or Alexa Fluor-647 were purchased from Jackson ImmunoResearch or Molecular Probes and used at 1:200 dilution. Sections incubated with biotinylated secondary antibody were rinsed and further incubated with streptavidin-Brilliant Violet 421 (1:200; BioLegend Cat# 405225) for 30 min. Slides stained without the fluorophore Brilliant Violet 421 were also counterstained with DAPI (4’,6-diamidino-2-phenylindole, Dilactate) (1 µg/ml; ThermoFisher Scientific Cat# D3571). Sections were coverslipped with Mowiol mounting medium or Vectashield mounting medium (Vector Laboratories, Burlingame, CA, USA) and subjected to fluorescence and confocal microscopic analysis. To identify newly generated oligodendrocytes following CNO delivery, mice were co-administered EdU (ThermoFisher Scientific Cat# E10415) at a final concentration of 50 mg/kg once daily via intraperitoneal injection. To assess EdU incorporation in mature oligodendrocytes and OPCs, sections were first processed for CC1/ASPA or PdgfRα immunohistochemistry as above, followed by EdU detection using the Alexa Fluor-594 Click-iT EdU Cell Proliferation Assay Kit (ThermoFisher Scientific Cat# C10339) according to the manufacturer’s instructions.

### Glial cell counts and myelin analysis

For all quantitative analyses, the investigator was blinded to each group allocation by assigning random numbers to each image file using the Filename_Randomizer macro for ImageJ.

Cell counts: Three to five coronal sections per mouse from the brain region encompassing the injection site (Bregma +0.38 mm to −0.34 mm)^47^ were used to quantify glial cell numbers. Likewise, a similar brain region was analyzed in electroporated mice. This brain region was independently confirmed to contain abundant decussating mCherry^+^ or GFP^+^ axons from the rAAV1/2-hM3Dq infected or in utero electroporated mice, respectively. The region of the CC spanning from one dorsolateral corner of the subventricular zone to the opposite side was used to analyze cell density, which was represented as the number of immunopositive cells divided by the area in mm^2^.

cFos analysis: For cFos analysis, three sections per mouse of the cortical region (Bregma +0.38 mm to −0.34 mm)^47^ where the majority of electroporated GFP^+^ neuron cell bodies were located were used. Images were sampled at 12-bit depth and collected using the same acquisition parameters to facilitate fluorescence intensity comparisons between groups. Fluorescence intensity was defined as (Fluorescence intensity = cell area × mean intensity−background intensity) and expressed as arbitrary units (AU).

Assessment of myelination: Sagittal sections were collected between the midline and +0.4 mm lateral to the midline^47^ in order to obtain transverse sections through decussating GFP^+^ axons in the CC. The region of the CC where the majority of GFP^+^ axons were decussating (posterior genu to anterior body, just superior to the fornix) was used for quantification. Three-to-five images per mouse were acquired using a 32-channel gallium arsenide phosphide (GaAsP) detector array. Scans from each channel were collected individually in multiple-track mode and subsequently merged and the density of MBP^+^ myelin rings, PAN-NF^+^ axons, and GFP^+^ axons was calculated by dividing the total number by the area analyzed. To calculate the myelinated fraction of GFP^+^ and PAN^+^ axons in an unbiased fashion, the FIJI colocalization plugin was used to segment out MBP^+^/GFP^+^ or MBP^+^/PAN^+^ axons. Briefly, the thresholded MBP^+^ rings were subjected to the “fill holes” function, and the resulting image was used as the colocalization template with a thresholded PAN^+^ or GFP^+^ channel. Only axons that were ≥80% colocalized with an MBP^+^ ring were counted as myelinated. Coronal sections were subjected to a similar analysis, with mGFP^+^/mCherry^+^ and mCherry^+^/PAN^+^ pixels segmented out with FIJI^48^. Only colocalized segments with ≥10 µm^2^ were used.

### Confocal microscopy

Stained coronal and sagittal sections were imaged by laser scanning confocal microscopy (LSM780, Zeiss), which was used to detect up to five fluorophores by laser excitation at 405, 488, 555, 594, and 633 nm wavelengths. Sections were typically captured at a magnification of 20× (PL-APO 20×/0.8 NA air objective) or 63× (PL-APO 63×/1.4 NA oil objective). Images were acquired using the same acquisition parameters for all samples in a single experiment and were saved as 8-bit LSM files for analysis with the NIH FIJI software (version 1.50i). All cell counts and analyses were performed blind to the experimental treatment.

### Immunoelectron microscopy

The protocol for immunolabeling the brain sections, amplification of the fluorescent signal, gold labeling, and subsequent silver enhancement of the gold were performed as previously described^49^. Briefly, P21 CNO-treated hM3Dq/GFP or GFP-control CD1 mice were transcardially perfused with 4% PFA (EM grade)/0.1% glutaraldehyde in PBS. Brains were postfixed for 1 h in 4% PFA/0.1% glutaraldehyde in HEPES-buffered saline (HBS) on ice and then overnight in 4% PFA in HBS at 4 °C. A Vibratome (VT1000P, Leica Microsystems GmbH) was used to cut 150 µm thick coronal sections, which were stored in 2.3 M sucrose in 0.1 M Sorensen’s phosphate buffer, pH 7.4, at 4 °C until processing. Permeabilization and blocking of nonspecific binding sites were performed by incubating free-floating sections with HBS plus 10% bovine serum albumin (BSA) and 0.05% Triton X-100 (Roche Diagnostics). Optimally titrated chicken anti-GFP antibody (GFP-1020, Aves Labs) was added at a final concentration of 5 µg/ml in a diluent of HBS plus 1% BSA and 0.005% Triton X-100 and incubated at 4 °C overnight. Following washing in PBS plus 0.05% BSA, sections were incubated with biotinylated goat anti-chicken IgY antibody (Vector Laboratories) at a concentration of 7.5 µg/ml for 30 min. Fluorescein tyramide amplification (PerkinElmer Life Sciences) for 6 min was used to amplify the signal. FITC was labeled with a mouse anti-FITC antibody conjugated to ultra-small gold (Aurion) for 2 h, and sections were postfixed in 2% PFA/2.5% glutaraldehyde in 0.1 M cacodylate buffer. Gold was silver enhanced with R-GENT SE-EM (Aurion) for 60 min. Sections were then extensively washed in distilled water, then fixed in 1% osmium tetroxide and 1.5% potassium ferrocyanide in distilled water. Subsequently sections were dehydrated using a graded series of alcohols, rinsed in acetone, and embedded in the resin of Spurr^50^. Prior to polymerization of the resin, the brain slices were trimmed to the areas of interest using a double-edged razor blade. Ultrathin sections (~90 nm) were cut with a Leica EM UC7 ultramicrotome. Contrast was developed using lead citrate and aqueous uranyl acetate prior to mounting sections on copper grids for transmission electron microscopy (TEM). As a negative control, brain sections from CD mice were labeled following the above protocol, excluding the primary antibody-labeling step.

Processed sections were examined in a JEOL 1011 TEM. MegaView III CCD cooled camera operated with iTEM AnalySIS software (Olympus Soft Imaging Systems GmbH) was used for image capture. Images were imported into FIJI to analyze the density of myelinated and unmyelinated GFP^+^ and GFP^−^ axons (10 images/mouse, *n* = 5 mice/group). *G*-ratios (axon diameter/diameter of the axon plus myelin sheath) were calculated using 100 GFP^+^ and 100 GFP^–^ axons per mouse (15–20 images/mouse; *n* = 5 mice/group). Images were analyzed with the researcher blinded to the experimental group. To avoid bias, the grid function in FIJI was used to randomly assign a region of axons for analysis.

### Generation of AAV1/2-sCAG-hM3Dq-mCherry viral vector

pAAV-sCAG-hM3Dq-mCherry-WPRE and Entry (pENTR) plasmids and pAAV-Gateway destination plasmid were a gift from Dr Melanie White (ARMI, Monash University, Clayton, VIC, Australia). pAAV-sCAG-hM3Dq-mCherry-WPRE was packaged into a rAAV mosaic serotype 1/2 capsid using the packaging plasmids pDPI and pDPII, as described^51^. Vectors were harvested and purified using iodixanol gradients. Viral titer (genomic copies (gc) per ml) was determined using quantitative polymerase chain reaction (qPCR). Briefly, reactions were conducted in a total volume of 25 µl containing 12.5 µl of SYBR Green Master Mix, 1 µl of forward primer (5’-CCGTTGTCAGGCAACGTG-3’, 10 µM), 1 µl of reverse primer (5’-AGCTGACAGGTGGTGGCAAT-3’, 10 µM), 5 µl of MgCl_2_ (25 mM), 0.5 µl of water, and 5 µl of either viral sample or standard. qPCR amplification of the WPRE transcriptonal element ^52^ was completed using an ABI Prism 7500HT Sequence Detection device (Applied Biosystems, Mulgrave, VIC, Australia) with conditions of 10 min at 95 °C followed by 40 cycles of denaturation at 95 °C of 15 s, annealing at 67 °C for 25 s, and extension/measurement at 72 °C for 25 s. Plasmid pAAV-sCAG-hM3Dq-mCherry-WPRE was used to produce the qPCR standard curve.

### In vivo stereotaxic injection

Postnatal day 5 (P5) mice were anesthetized by inducing hypothermia^53^. Briefly, pups were placed in a box of crushed ice for 3–5 min, which typically yielded a period of torpor for up to 15 min. We placed the pups on a piece of surgical drape to avoid direct damage to the skin. Pups then received a unilateral 1.0 µl injection of rAAV1/2-sCAG-hM3Dq-mCherry-WPRE (1.0 × 10^11^ viral gc/ml) into the right S1 cortex (~2.10 mm lateral of the sagittal suture, ~3.50 mm anterior of the lambdoid transverse suture). A modified stereotaxic frame stage was made to account for the smaller size of mouse neonates^54^.

### Electrophysiology and acute brain slice preparation

To make cortical slices, adult (*n* = 4) C57Bl/6 female mice were deeply anesthetized with 5% w/v isoflurane, the forebrain removed, rapidly cooled, and trimmed to yield a tissue block approximately centered on the viral injection site. Coronal slices (200 µm) were cut with a vibrating microtome (VT1200S; Leica Microsystems Inc., Bannockburn, IL, USA). The external solution was an artificial cerebrospinal fluid (aCSF) containing (mM): 125 NaCl, 3 KCl, 1.2 KH_2_PO_4_, 1.2 MgSO_4_, 25 NaHCO_3_, 10 dextrose, and 2 CaCl_2_. Slices were secured with a nylon mesh in a tissue chamber and perfused with aCSF at 34 °C, 300 mOsm, bubbled with 95% O_2_–5% CO_2_.

For whole-cell recordings, recording pipettes (3.2–4.0 MΩ) were guided to AAV1/2-sCAG-hM3Dq-mCherry-expressing (*n* = 5 cells), and non-expressing pyramidal neurons (*n* = 5 cells) in the layer 2/3 of the cortex. Neurons were visualized for patching with dodt contrast optics (40 × water immersion lens) on an Axio Examiner fixed stage microscope (Zeiss, Thornwood, NJ) with digital camera (Rolera EM-C^2^, Q imaging, Surrey, BC, Canada). Pipettes were filled with an intracellular solution containing (mM): 10 NaCl, 40 KCl, 90 K-gluconate, 11 EGTA, 1 CaCl_2_, 1 MgCl_2_, 10 HEPES, 2 Na_2_ATP, and 0.2 Na_2_GTP and 0.5% biocytin (pH 7.3 and 290 mOsm). All recordings were made in open, whole-cell patch configuration under voltage or current clamp using a Multiclamp 700B amplifier (Molecular Devices, Sunnyvale, CA, USA). Signals were sampled at 20 kHz and filtered at 10 kHz using the p-Clamp software (version 10.3, Molecular Devices, Sunnyvale, CA, USA). Liquid junction potentials were corrected for during data analysis (10.2 mV at 34 °C).

CNO (1 µM, Tocris Bioscience, Bristol, UK) was bath applied for 5–7 min and subsequently washed in external solution.

All recorded neurons were processed immunohistochemically to label mCherry and biocytin to confirm groups.

### Data analysis and statistics

Membrane potential (Vm) was measured over the immediate 2 min prior to CNO application and defined as baseline. The peak change in Vm over 2 min was measured 5–10 min after the beginning of CNO exposure. Comparisons were made by one-way repeated-measures ANOVA with Dunnett post-hoc test. For input–output curves, action potentials were counted over the step period and groups compared by two-way ANOVA with Bonferroni post-hoc (SigmaStat, San Jose, CA, USA).

### Analysis of oligodendrocyte production

To calculate the number of post-mitotic oligodendroglia that are likely to have been generated between P60 and P60+7, we made the following assumptions, based on previous reports: (1) OPC density in the CC of non-stimulated (GFP-only) control mice at P60 is equivalent to that observed at P60+14 (219 cells/mm^2^) on the basis that OPC density is under tight homeostatic control^33^. (2) That 82% of mitotic OPCs undergo asymmetric division in the CC of the adult mouse brain, with each asymmetric mitosis giving rise to one OPC and one post-mitotic cell, based upon the asymmetric localization of epidermal growth factor receptor on a single daughter cell within OPC cell pairs isolated from the adult CC^55^. (3) The cell cycle time of OPCs at P60 is 9.5 days^22^. For simplicity, we consider the contribution of asymmetric mitoses in isolation and calculate that these 219 OPCs/mm^2^ would be expected to generate ~300 post-mitotic oligodendroglia/mm^2^ over the 7 day labeling period in the control mice, based on the equation *N*_P60+14_ = *N*_P60_2^(*t*/*d*)^ where *N*_P60_ = no. of asymmetrically dividing OPCs at P60 (219 × 0.82); time, *t* = 7 days and cell cycle time, *T*_c_ = 9.5 days. At P60+14, we identified 20 EdU^+^/ASPA^+^ cells/mm^2^ (Fig. 3f); however, this only accounts for a fraction of new oligodendrocytes that would have been generated during this period since EdU was administered once daily. Assuming EdU bioavailability is ~2 h in the adult brain, based on that of the thymidine analog bromodeoxyuridine^56, 57^, we estimate that a single intraperitoneal injection of EdU labels 1/12th (2 h/24 h) of new oligodendrocytes that are generated per day. On this basis, we conservatively estimate that the actual number of new oligodendrocytes generated over the 7-day EdU labeling period is ~240 cells/mm^2^ (12 × 20). In adult stimulated mice, we identified 35 EdU^+^/ASPA^+^ cells/mm^2^ at P60+14 (Fig. 3f) and calculate that this reflects the generation of ~420 oligodendrocytes/mm^2^ (12 × 35) during the 7-day labeling period.

### Statistical analysis

All statistical analyses were performed using the GraphPad Prism software (ver. 7.0). Statistical significance was determined using unpaired, two-tailed Student’s *t*-tests with Welch’s correction, one-way ANOVA, or two-way ANOVA with Tukey’s post-hoc multiple-comparison tests. Statistical significance was defined as *p* < 0.05. Quantitative data are reported as mean ± s.e.m.

### Data availability

The data that support the findings of this study are available from the corresponding authors upon reasonable request.

## Electronic supplementary material

Supplementary Information
