# Supplementary material for: Pharmacogenetic stimulation of neuronal activity increases myelination in an axon-specific manner
Source: Nat Commun. 2018 Jan 22;9:306. doi: 10.1038/s41467-017-02719-2 (PMC5778130; doi:10.1038/s41467-017-02719-2)
Supplement: Supplementary file 1 — Supplementary Information [file 41467_2017_2719_MOESM1_ESM.pdf]

Supplementary Information

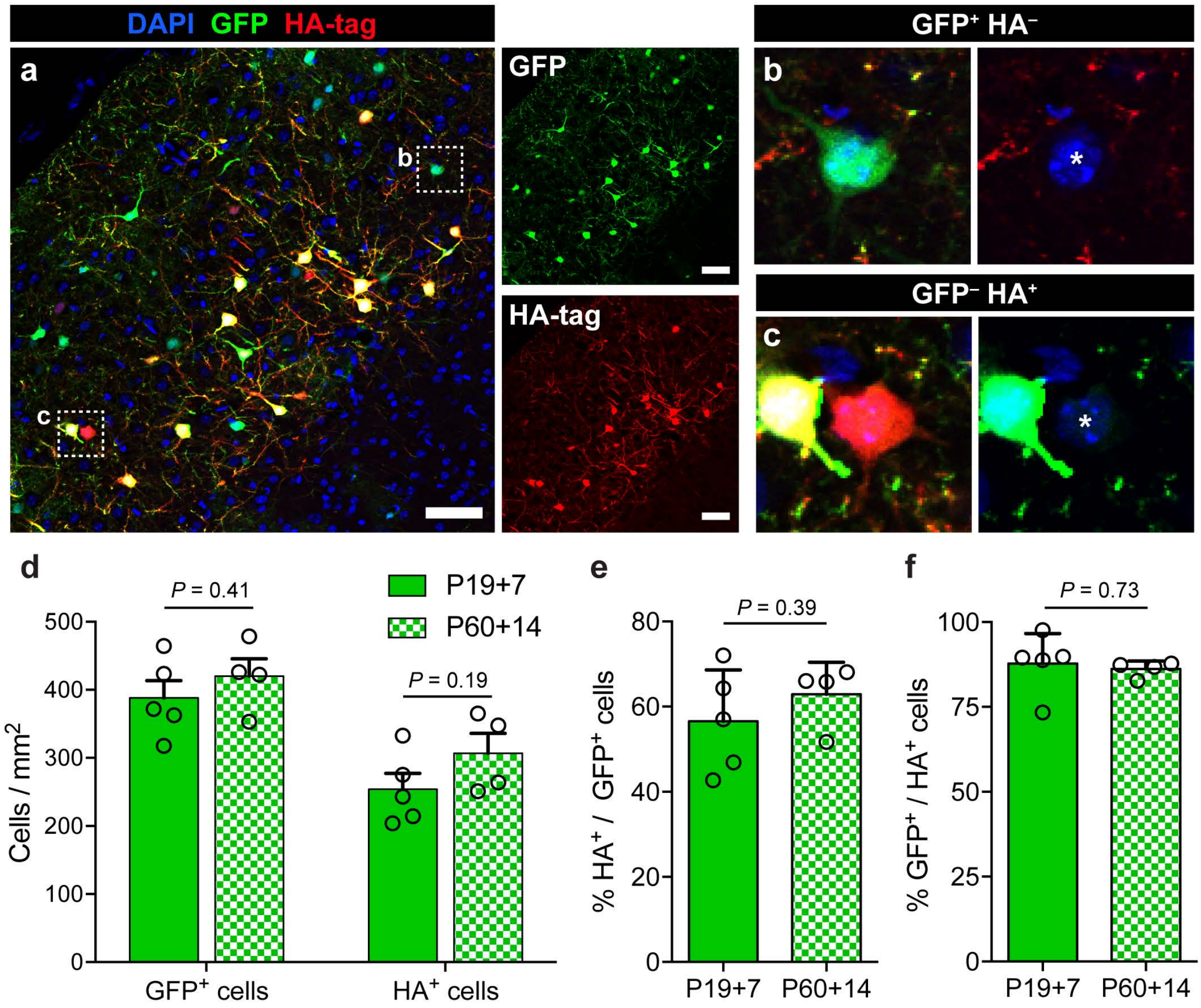

Supplementary Figure 1. Analysis of HA-hM3Dq and Gfp plasmid co-electroporation efficiency. Related to Figure 1.

(**a**) Representative image of the right S1 showing *Gfp* (inset right: top) and *HA-hM3Dq* (inset right: bottom) co-electroporated layer 2/3 pyramidal neurons. (**b,c**) Enlargement of boxed regions from (**a**) depicting examples of a $GFP^{+}HA^{-}$ (**b**) or $GFP^{-}HA^{+}$ (**c**) neurons. (**d**) Quantification of the density of GFP^{+} and HA^{+} neurons in either juvenile (P19+7) or adult (P60+14) mice. There was no appreciable loss of expression of either plasmid with age. (**e,f**) Analysis of the proportion of GFP^{+} neurons that co-expressed HA-tag (**e**), and vice versa, (**f**) the proportion of HA^{+} neurons that co-expressed GFP. Welch's corrected unpaired two-tailed *t*-test: *n*=4-5 mice/condition, ± s.e.m. Scale bars = 50 μm

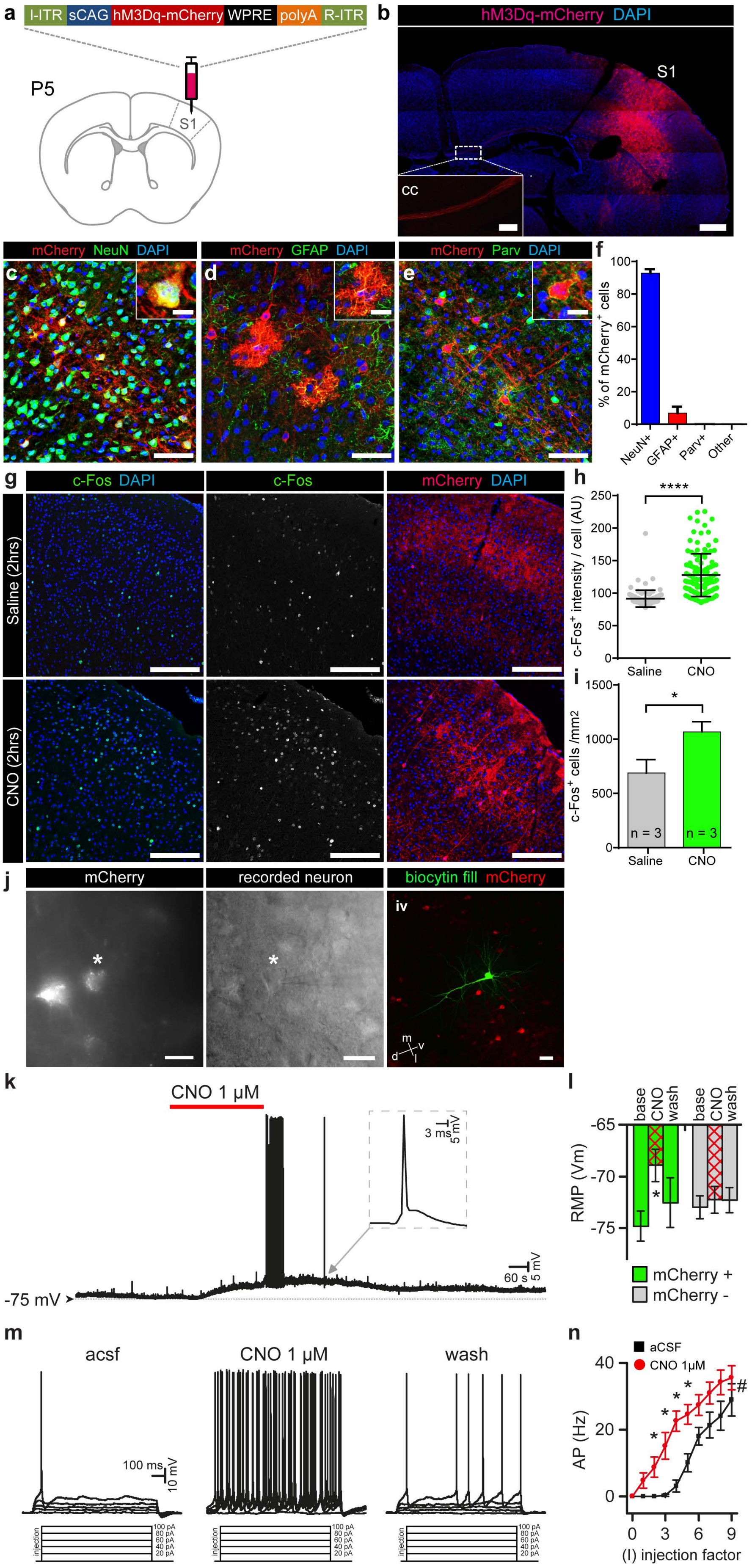
a
l-ITR sCAG hM3Dq-mCherry WPRE polyA R-ITR
P5
S1
b
hM3Dq-mCherry DAPI
S1
cc
mCherry NeuN DAPI
c
mCherry GFAP DAPI
d
mCherry Parv DAPI
e
f
% of mCherry+ cells
NeuN+
GFAP+
Parv+
Other
g
c-Fos DAPI
c-Fos
mCherry DAPI
Saline (2hrs)
CNO (2hrs)
h
c-Fos+ intensity / cell (AU)
****
Saline
CNO
i
c-Fos+ cells /mm2
*
n = 3
n = 3
Saline
CNO
j
mCherry
recorded neuron
biocytin fill mCherry
iv
k
CNO 1 μM
3 ms
5 mV
60 s
5 mV
-75 mV
l
base
CNO
wash
base
CNO
wash
RMP (Vm)
mCherry +
mCherry -
m
acsf
CNO 1 μM
wash
100 ms
10 mV
injection
100 pA
80 pA
60 pA
40 pA
20 pA
n
aCSF
CNO 1μM
AP (Hz)
(I) injection factor

Supplementary Figure 2. Electrophysiological characterization of the hM3Dq DREADD. Related to Figure 1.

(**a**) hM3Dq-mCherry was packaged into an adeno-associated viral vector (AAV1/2-CAG-hM3Dq-mCherry), which was injected into the right somatosensory cortex of postnatal day 5 mice. (**b**) Representative image of mCherry expression throughout S1 cortex and callosal axons at P20. (**c-f**) Representative images of P20 cortex immunolabeled with mCherry and neuronal marker NeuN (**c**), mCherry and astrocyte marker GFAP (**d**) or mCherry and interneuron marker Parvalbumin (**e**). (**f**) Proportion of cortical cells that expressed mCherry and another cellular marker. There were no mCherry expressing cells in the corpus callosum (data not shown). (**g**) Representative images of cFos labeling in the right S1 cortex 2 hours after saline (top panels) or CNO (bottom panels) administration. (**h,i**) Analysis of cFos fluorescence intensity (**h**) and number of $cFos^+$ cells (**i**) in the S1 cortex where the majority of hM3Dq-$mCherry^+$ cells are localized (Welch's corrected unpaired two-tailed *t*-test: $*P < 0.05$, $****P < 0.0001$, $n=3$ mice/condition). (**j**) Representative images of cortical slices used for electrophysiological characterization. Neurons expressing hM3Dq were identified by mCherry fluorescence and patched for recording after which they were filled with biocytin to confirm their pyramidal identity. (**k**) Example recording from a patched $hM3Dq^+$ pyramidal neuron showing depolarization following exposure to 1 µM CNO. (**l**) Analysis of resting membrane potential in patched $hM3Dq^+$ and $hM3Dq^-$ neurons following CNO exposure and washout ($P = 0.025$, $n=5$ cells, Two-way ANOVA with Bonferroni post-hoc test). (**m,n**) Three out of five $hM3Dq^+$ pyramidal neurons exhibited recurrent bursting following 1 µm CNO exposure (**m**) while those that did not, required less excitation (current injection) to fire action potentials (APs) when depolarized by CNO as seen by a significant leftward shift in the input-output relationship (**n**). $P < 0.001$, $n=5$ cells, Two-way ANOVA with Bonferroni post-hoc test, ± s.e.m. Scale bars = 200 µm (**b,g**), 50 µm (**b** insert**, c,d,e**), 20 µm (**j**), 10 µm (**c,d,e** insert).

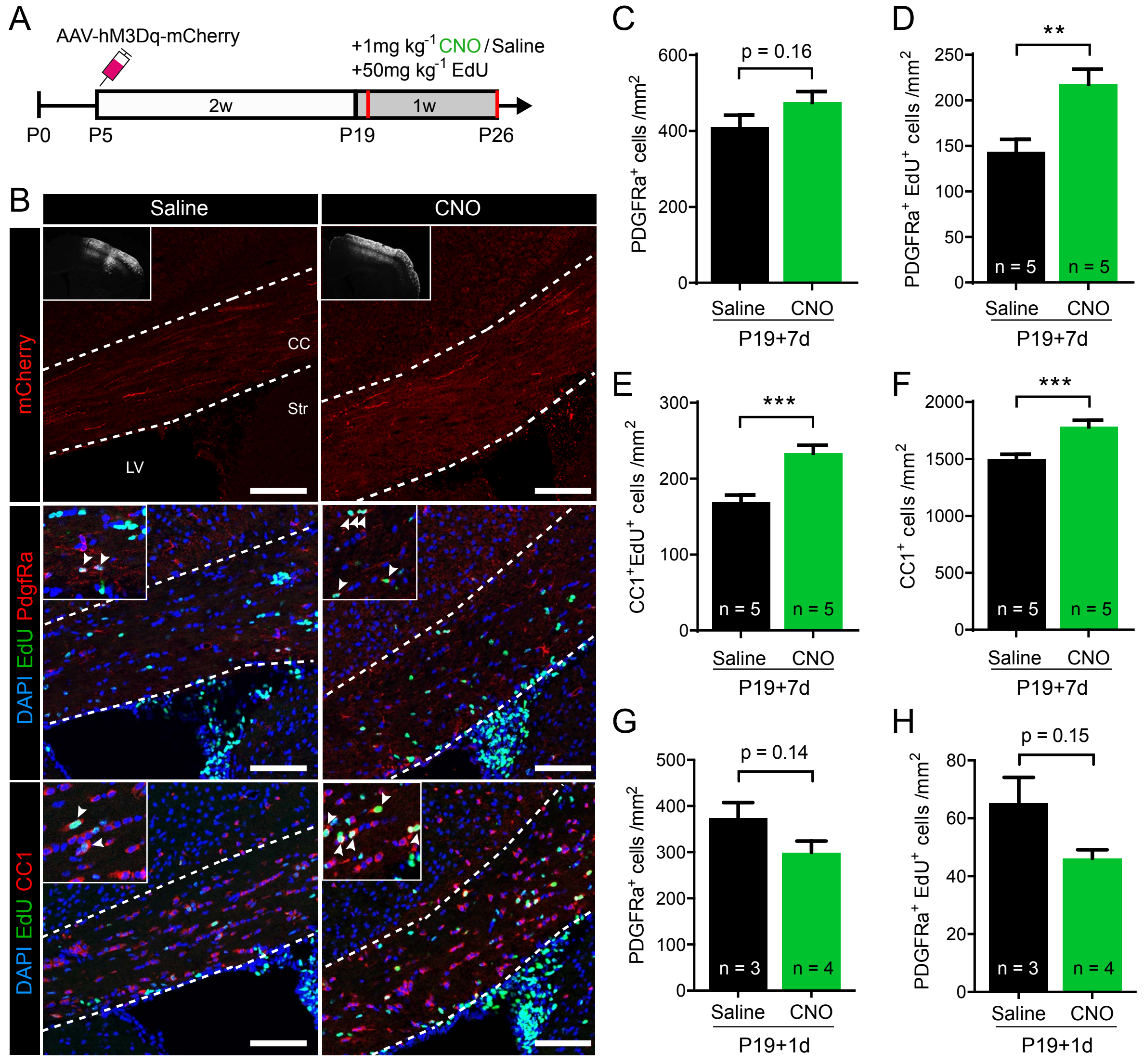

Supplementary Figure 3. DREADD-mediated neuronal stimulation in juvenile mice results in increased differentiation of oligodendrocyte progenitor cells. Related to Figure 2.

(**a**) C57Bl/6 mice expressing rAAV1/2-hM3Dq-mCherry received EdU in conjunction with either CNO or saline vehicle once daily, and were sacrificed 1 or 7 days later (day of perfusion depicted by red vertical lines). (**b**) Representative images from coronal sections of the corpus callosum encompassing the mCherry^{+} axons (top panel) of hM3Dq expressing mice administered saline or CNO for one week. Insets demonstrate the higher proportion of EdU^{+}/PdgfRα^{+} OPCs (white arrowheads) and EdU^{+}/CC1^{+} newly-differentiated oligodendrocytes (white arrowheads) in CNO-challenged mice in the middle and bottom panels, respectively. (**c**,**d**) Analysis of total density of OPCs (**c**) and dividing OPCs (**d**) at 1 week post-stimulation. (**e**,**f**)

Quantification of the density of mature oligodendrocytes revealed an increase in newly differentiated oligodendrocytes (**e**), as well as total oligodendrocyte density (**f). (g,h**) At one day post-stimulation, there was no difference in either the total OPC density (**g**) or density of dividing OPCs (**h**) between CNO stimulated and vehicle treated control mice. Welch's corrected unpaired two-tailed *t*-test: $^*P < 0.05$, $^{**}P < 0.01$, $^{***}P < 0.001$, ± s.e.m. Scale bars = 50 μm.

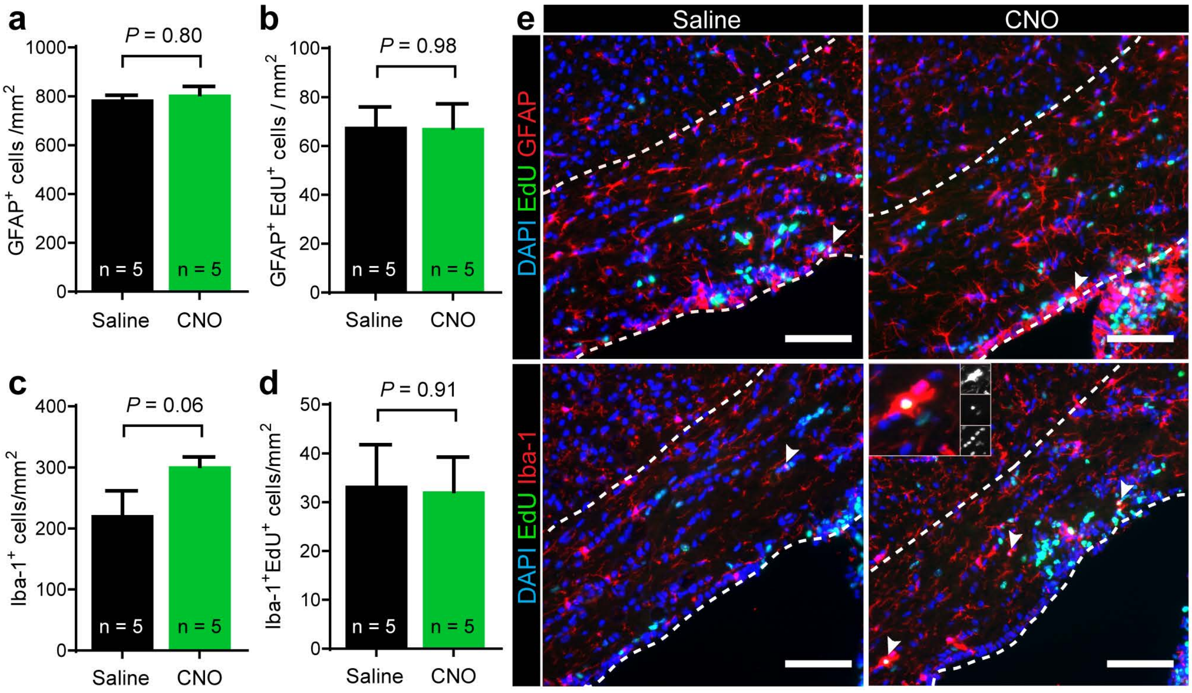

Supplementary Figure 4. DREADD-mediated neuronal stimulation in juvenile mice does not affect the astroglial or microglial population. Related to Figure 2. (**a**,**b**) Quantification of the density of total (**a**) and dividing (**b**) $GFAP^+$ astrocytes in the corpus callosum of C57Bl/6 mice expressing rAAV1/2-hM3Dq-mCherry following one week treatment with CNO or saline. (**c**,**d**) The density of total (**c**) and dividing (**d**) Iba-1^+ microglia were also quantified. There was no difference in either glial population between CNO stimulated mice and vehicle treated controls. (**e**) Representative sections of the corpus callosum showing similar levels of microglial and astroglial marker labeling. Welch's corrected unpaired two-tailed *t*-test: *n*=5 mice/condition, ± s.e.m. Scale bars = 100 µm.

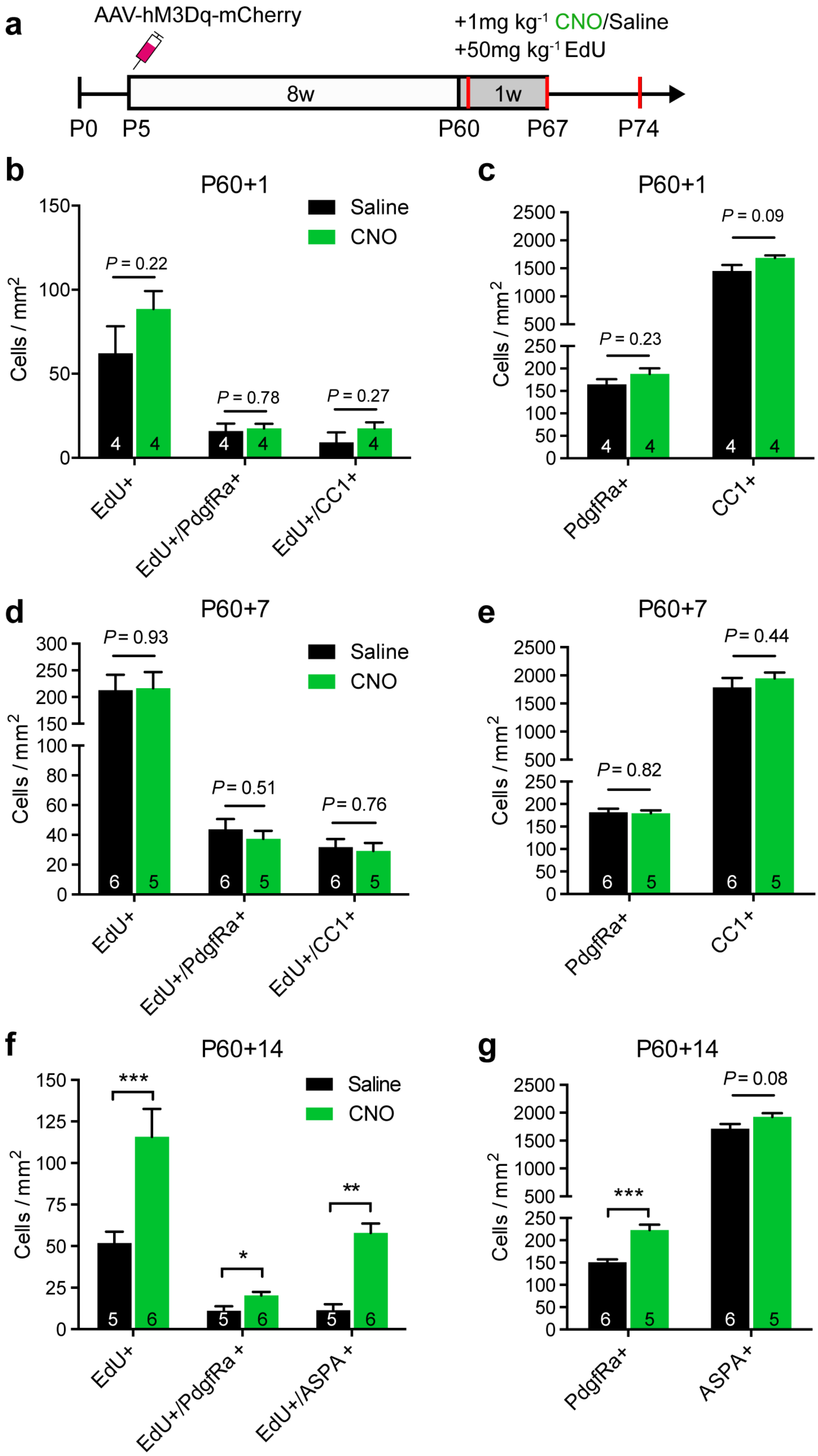
a
AAV-hM3Dq-mCherry
+1mg kg-1 CNO/Saline
+50mg kg-1 EdU
8w
1w
P0
P5
P60
P67
P74
b
P60+1
Saline
CNO
Cells / mm2
P = 0.22
P = 0.78
P = 0.27
EdU+
EdU+/PdgfRa+
EdU+/CC1+
c
P60+1
P = 0.09
P = 0.23
PdgfRa+
CC1+
d
P60+7
P= 0.93
P= 0.51
P= 0.76
e
P60+7
P= 0.44
P= 0.82
f
P60+14
***
*
**
EdU+/ASPA+
g
P60+14
ASPA+

Supplementary Figure 5. DREADD-mediated neuronal stimulation in adult mice results in increased proliferation and differentiation of oligodendrocyte progenitor cells. Related to Figure 3.

(**a**) P60 C57Bl/6 mice expressing rAAV1/2-hM3Dq-mCherry received EdU in conjunction with either CNO or saline vehicle daily before being sacrificed 1, 7 or 14 days later (day of perfusion depicted by red vertical lines). (**b,c**) The density of mitotic cells (**b**) and total OPCs and oligodendrocytes (**c**) after one day of CNO/EdU treatment was not significantly different. (**d,e**) Analysis of the density of dividing OPCs and newly-differentiated oligodendrocytes (**d**) revealed no difference between stimulated and vehicle groups, resulting in no net change in total oligodendroglial lineage cells (**e**) following a week of CNO administration. (**f,g**) Quantification of the total density (**g**) and density of dividing (**f**) OPCs and oligodendrocytes at P60+14. There was a significant increase in the density of newly-differentiated $EdU^+/ASPA^+$ oligodendrocytes (**f**) but no change in the total density of mature oligodendrocytes in stimulated animals compared to vehicle controls (**g**). Welch's corrected unpaired two-tailed *t*-test: *$P < 0.05$, **$P < 0.01$, ***$P < 0.001$, ± s.e.m.

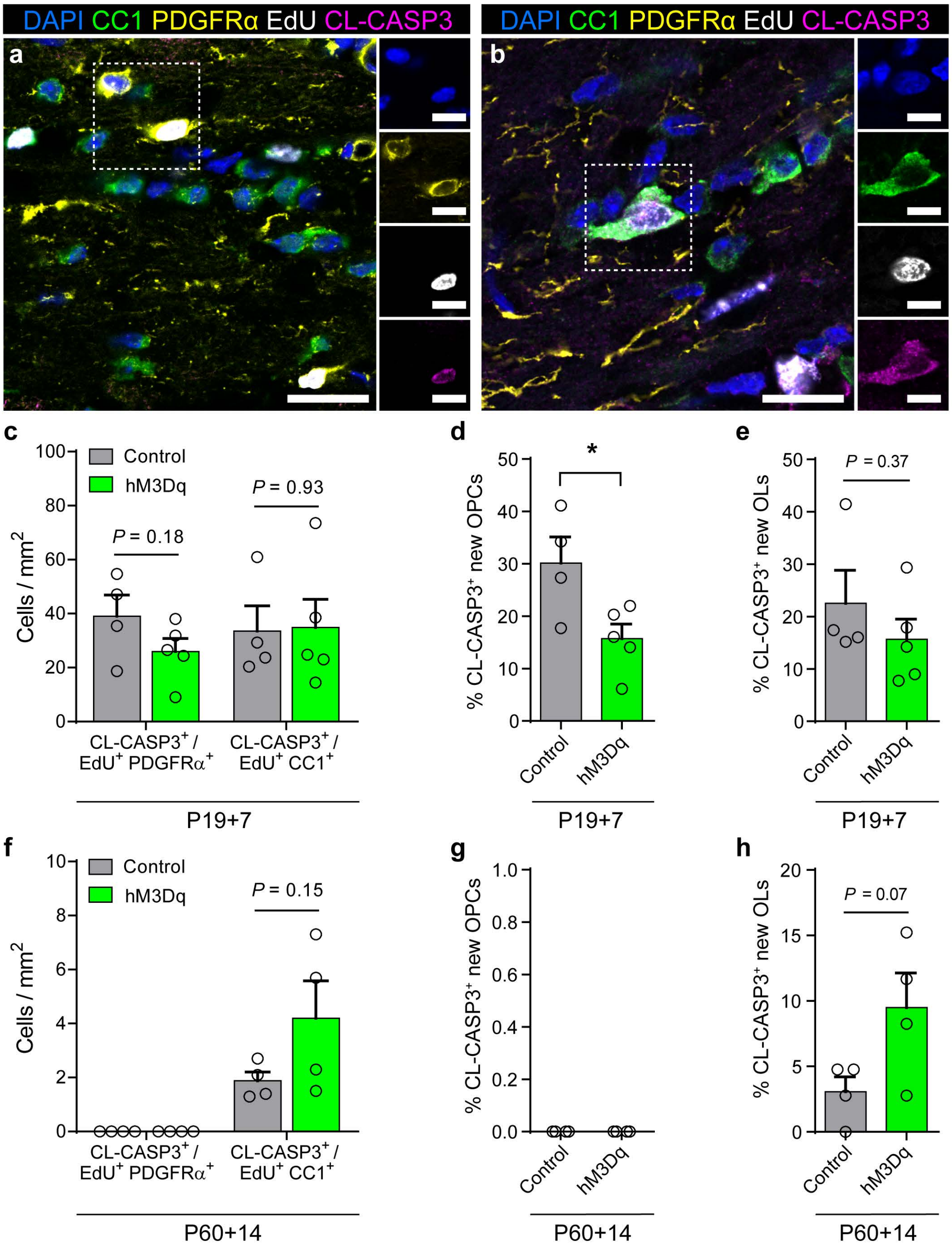

Supplementary Figure 6. DREADD-mediated neuronal stimulation had a pro-survival effect in juvenile but not adult mice. Related to Figures 2 and 3.

(**a,b**) Representative images of cleaved caspase-3 (CL-CASP3) activity in cycling OPCs (**a**) or newly-differentiated (EdU+) oligodendrocytes (**b**). Insets depict a higher magnification of the boxed region from each image. (**c**)

Quantification of the density of CL-CASP3$^+$/EdU$^+$ OPCs and oligodendrocytes in juvenile mice. (**d,e**) Analysis of the percentage of newly-generated OPCs (**d**) or oligodendrocytes (**e**) that were marked for cell death in juvenile mice. (**f**) Quantification of the density of CL-CASP3$^+$/EdU$^+$ OPCs and oligodendrocytes in adult mice. (**g,h**) Analysis of the percentage of newly-generated OPCs (**d**) or oligodendrocytes (**e**) that were CL-CASP3$^+$ in adult mice. Welch's corrected unpaired two-tailed *t*-test: *$P < 0.05$, n=4-5 mice/condition, ± s.e.m. Scale bars = 20 μm.

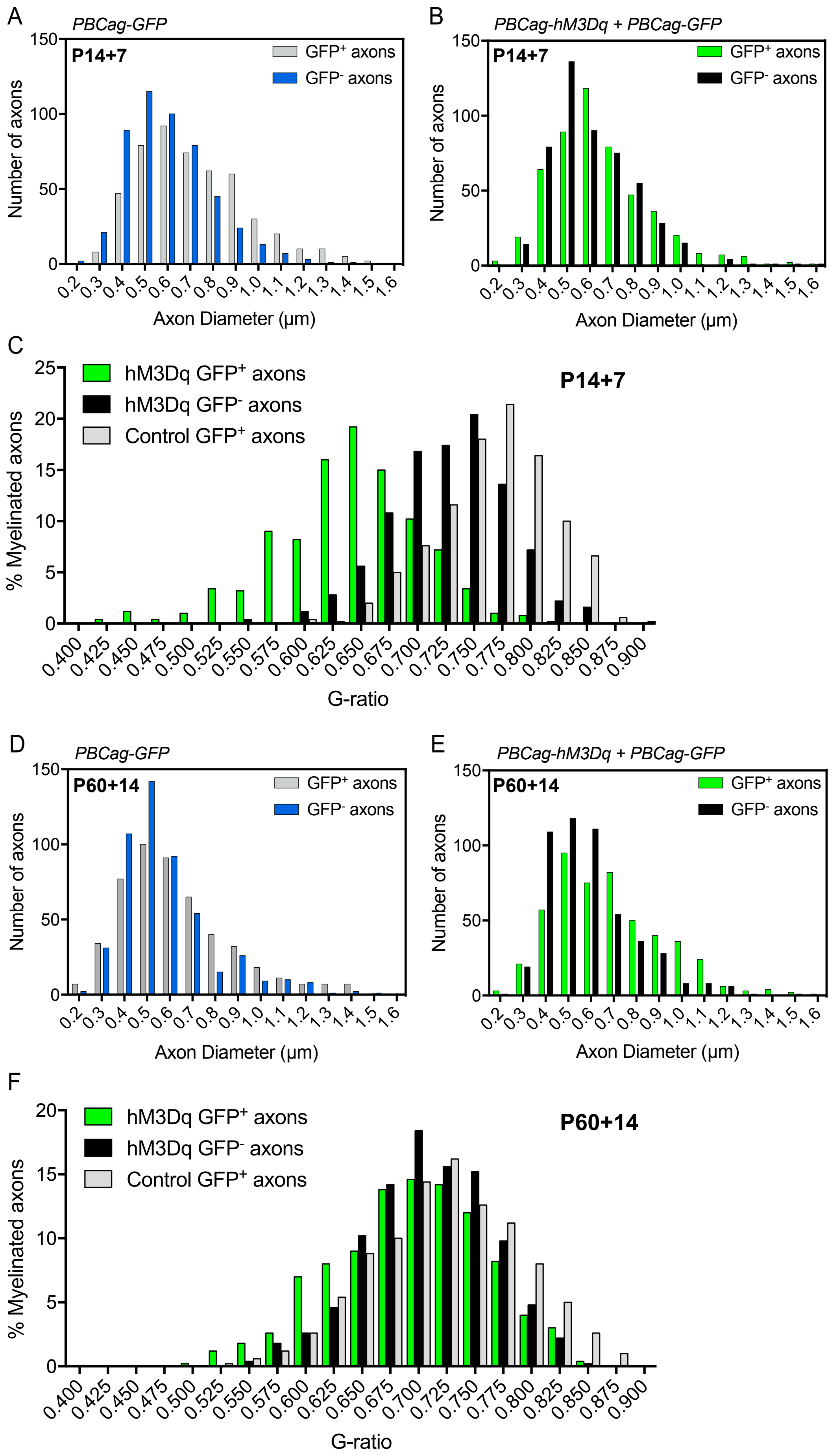

A
PBCag-GFP
P14+7
GFP+ axons
GFP- axons
Number of axons
Axon Diameter (µm)
B
PBCag-hM3Dq + PBCag-GFP
P14+7
GFP+ axons
GFP- axons
Number of axons
Axon Diameter (µm)
C
hM3Dq GFP+ axons
hM3Dq GFP- axons
Control GFP+ axons
P14+7
% Myelinated axons
G-ratio
D
PBCag-GFP
P60+14
GFP+ axons
GFP- axons
Number of axons
Axon Diameter (µm)
E
PBCag-hM3Dq + PBCag-GFP
P60+14
GFP+ axons
GFP- axons
Number of axons
Axon Diameter (µm)
F
hM3Dq GFP+ axons
hM3Dq GFP- axons
Control GFP+ axons
P60+14
% Myelinated axons
G-ratio

Supplementary Figure 7. DREADD-mediated activity stimulation results in reduced g-ratios in the absence of an effect on axonal diameter. Related to Figures 6 and 7.

(**a,b**) The frequency distribution of myelinated axons from the developing (P14+7) corpus callosum of GFP control (**a**) and hM3Dq/GFP (**b**) mice. (**c**) Activity stimulation for one week resulted in a leftwards shift in the *g*-ratio (thicker myelin) of the entire hM3Dq axon population studied compared to both GFP^+ axons in the controls, as well as adjacent GFP^- axons within the same sections. (**d,e**) The frequency distribution of myelinated axons from the adult (P60+14) corpus callosum of GFP control (**d**) and hM3Dq/GFP (**e**) mice. Axons were randomly picked for *g*-ratio analysis (*n*=100 axons/type/mouse; 5 mice/condition) and displayed a pattern approaching normal distribution in axon size with axons of 0.4-0.7 µm diameter being the most abundant. (**f**) In adult mice, activity stimulation produced a leftwards shift in the $hM3Dq/GFP^+$ axon population, albeit to a much smaller extent than in young mice.
